# Targeting TOMM40 and TOMM22 to Rescue Statin-Impaired Mitochondrial Function, Dynamics, and Mitophagy in Skeletal Myotubes

**DOI:** 10.3390/ijms262210977

**Published:** 2025-11-13

**Authors:** Neil V. Yang, Sean Rogers, Rachel Guerra, Justin Y. Chao, David J. Pagliarini, Elizabeth Theusch, Ronald M. Krauss

**Affiliations:** 1 Department of Nutritional Sciences & Toxicology, University of California, Berkeley, CA 94704, USA; 2 Department of Pediatrics, University of California, San Francisco, CA 90048, USA; 3 Department of Cell Biology and Physiology, Washington University School of Medicine, St. Louis, MO 63110, USA; 4 Department of Biochemistry and Molecular BioPhysics, Washington University School of Medicine, St. Louis, MO 63110, USA; 5 Department of Genetics, Washington University School of Medicine, St. Louis, MO 63110, USA; 6 Department of Medicine, University of California, San Francisco, CA 90048, USA

**Keywords:** statin, mitochondrial dynamics, skeletal muscle, translocase of outer mitochondrial membrane, transmission electron microscopy

## Abstract

Statins are the drugs most commonly used for lowering plasma low-density lipoprotein (LDL) cholesterol levels and reducing cardiovascular disease risk. Although generally well-tolerated, statins can induce myopathy, a major cause of non-adherence to treatment. Impaired mitochondrial function has been implicated in the development of statin-induced myopathy, but the underlying mechanism remains unclear. We have shown that simvastatin downregulates the transcription of *TOMM40* and *TOMM22*, genes that encode major subunits of the translocase of the outer mitochondrial membrane (TOM) complex. Mitochondrial effects of knockdown of *TOMM40* and *TOMM22* in mouse C2C12 and primary human skeletal cell myotubes include impaired oxidative function, increased superoxide production, reduced cholesterol and CoQ levels, and disrupted markers of mitochondrial dynamics and morphology as well as increased mitophagy, with similar effects resulting from simvastatin exposure. Overexpression of *TOMM40* and *TOMM22* in simvastatin-treated mouse and human skeletal muscle cells rescued effects on markers of mitochondrial dynamics and morphology, but not oxidative function or cholesterol and CoQ levels. These results show that TOMM40 and TOMM22 have key roles in maintaining both mitochondrial dynamics and function and indicate that their downregulation by statin treatment results in mitochondrial effects that may contribute to statin-induced myopathy.

## 1. Introduction

Statins, the most widely used class of drugs for reducing plasma LDL-cholesterol levels and cardiovascular disease risk, act by inhibiting 3-hydroxy-3-methyglutaryl coenzyme A reductase (HMGCR), the rate-limiting enzyme for cholesterol synthesis [1,2]. Although statins are highly effective and generally well-tolerated, they can have adverse side effects, the most common being statin-associated muscle symptoms (SAMS) [3,4] ranging from myalgia and myositis to rhabdomyolysis [5]. Among the currently used statins, simvastatin has been associated with the greatest incidence of SAMS [6].

Disruption of mitochondrial function has been proposed as a major mechanism contributing to SAMS [7]. Effects of statins on skeletal muscle mitochondria phenotypes have been demonstrated in cellular, animal, and clinical studies [8,9]. These include increased reactive oxygen species (ROS) [10], decreased mitochondrial biogenesis [11], altered protein prenylation, decreased intracellular ATP levels [12,13], altered electron transport chain protein expression, increased fragmentation of mtDNA [14], and elevated plasma creatine kinase (CK) levels [15]. Additionally, statin-induced inhibition of the synthesis of coenzyme Q (CoQ_10_), an essential cofactor of the electron transport chain, has been proposed as a major contributing factor to skeletal muscle mitochondrial dysfunction [16]. Despite these findings, little is known of the molecular basis for these statin effects.

**Figure 1:**
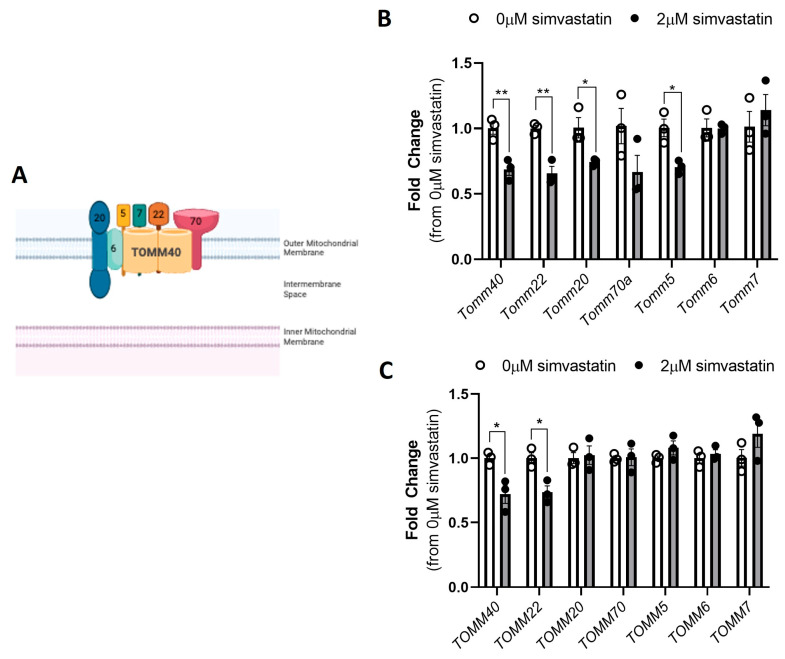
Simvastatin downregulates *TOMM40* and *TOMM22* in both C2C12 and hSkMC skeletal muscle. Differentiated C2C12 and primary hSkMC myotubes were treated with 2 µM simvastatin for 24 h. (**A**) A schematic diagram of the mammalian TOM complex, consisting of seven subunits, located in the outer mitochondrial membrane. (**B**) Simvastatin treatment (2 µM) downregulates *Tomm40*, *Tomm22*, *Tomm20*, and *Tomm5* in differentiated C2C12 myotubes. (**C**) Simvastatin treatment (2 µM) downregulates *TOMM40* and *TOMM22* in hSkMC myotubes. Numeric data represent the mean ± SEM. * *p* < 0.05, ** *p* < 0.01 vs. 0 µM simvastatin by the Student’s *t*-test (*n* = 3 biological replicates).

Recently, Grunwald et al. reported that simvastatin exposure of myotubes derived from primary human myoblasts resulted in a significant 50% reduction in expression of *TOMM40* and *TOMM22* [17], two broadly expressed and highly conserved genes encoding components of the translocase of the outer mitochondrial membrane (TOM) complex [18]. We have observed a similar effect of simvastatin in a panel of human lymphoblastoid cell lines [19]. *TOMM40* and *TOMM22* were among the top 22 transcripts downregulated by simvastatin treatment. The mammalian TOM complex consists of seven subunits that work together to recognize and import proteins from the cytoplasm into the mitochondrial interior to maintain mitochondrial function [20,21] (Figure 1A). Among these subunits, TOMM40 is the main channel-forming subunit that is stably associated with TOMM22, the central receptor of the complex [22]. Together, they are actively involved in protein translocation across the mitochondrial outer membrane [23]. We have previously shown in hepatocytes that TOMM40 plays a central role at mitochondria-ER contact sites (MERCs) in regulating hepatocellular cholesterol and lipid metabolism [24]. In addition, TOMM40 is known to interact with several ER membrane proteins at mitochondria-ER contact sites (MERCs) including B cell receptor-associated protein 31 (BCAP31), which has been shown to regulate cell death and crosstalk signaling between ER and mitochondria [25,26], while TOMM22 binds with PINK1 to eliminate damaged mitochondria via mitophagy [27].

In the present study, we performed detailed mitochondrial phenotyping to compare the effects of *TOMM40* and *TOMM22* knockdown with the effects of simvastatin treatment in two mammalian skeletal muscle cell models—mouse C2C12 cells, which are known to display cellular phenotypes with statin treatment similar to those seen with statin-induced myotoxicity in humans [28], and primary human skeletal muscle cells (hSkMCs). We also tested the role of *TOMM40* and *TOMM22* downregulation in mediating statin’s mitochondrial effects by determining whether these effects were rescued by overexpressing these genes. Finally, we showed that the transcription of *BCAP31* was reduced by both statin treatment and *TOMM40* knockdown, and therefore tested whether reduced *TOMM40* expression may play a role in mediating the mitochondrial effects of statin in skeletal muscle cells.

## 2. Results

### 2.1. Simvastatin Downregulates Key Subunits of the TOM Complex in Mammalian Skeletal Muscle Cells

We first aimed to confirm previous findings in primary human myotubes [23] that the expression of *TOMM40* and *TOMM22* was downregulated by simvastatin exposure. Differentiated C2C12 and primary hSkMC myotubes were treated with 2 µM simvastatin for 24 h. This dose was chosen based on simvastatin dose response experiments that showed significant induction of *Hmgcr* and *Ldlr* mRNA at 2 µM in C2C12 cells (Figure S1). Additionally, we performed a simvastatin dose-response experiment in C2C12 cells to assess apoptosis using EarlyTox Caspase-3/7 and found no significant increase with simvastatin 2 µM vs. the baseline (Figure S2). In both cell types, mRNA transcript levels of *TOMM40* and *TOMM22* were significantly reduced by exposure to 2 µM simvastatin as assessed by qRT-PCR (Figure 1B,C). We also confirmed significant reductions in TOMM40 and TOMM22 protein levels in primary hSkMC myotubes exposed to 2 µM simvastatin by Western blot (Figure S3). Transcript levels of two other subunits of the TOM complex, *Tomm20* and *Tomm5*, were also significantly reduced by simvastatin in the C2C12 myotubes but not primary hSkMC myotubes (Figure 1B,C). These results confirm and extend the evidence that simvastatin downregulates the expression of major subunits of the TOM complex.

### 2.2. TOMM40 and TOMM22 Knockdown Impairs Mitochondrial Function in Skeletal Myotubes

**Figure 2:**
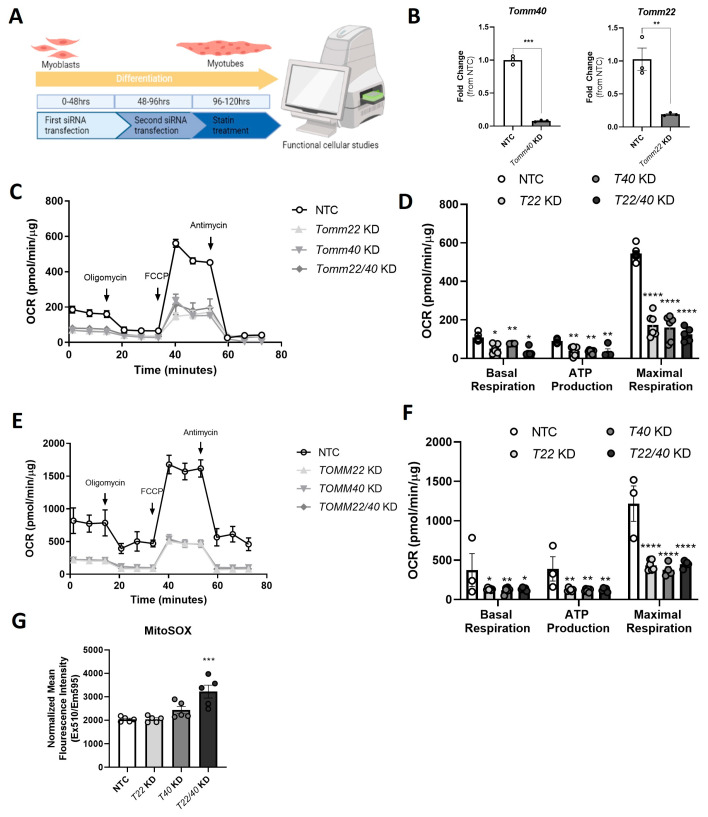
*TOMM40* and *TOMM22* knockdown decreases the mitochondrial oxygen consumption rate and promotes mitochondrial superoxide production. (**A**) Schematic illustration of the two-step transfection and differentiation experiment in C2C12 cells in vitro. (**B**) Confirmation of *Tomm40* (T40) and *Tomm22* (T22) KD in C2C12 myotubes by ~92% and ~85%, respectively, measured with qPCR (*n* = 3 biological replicates). (**C**) Oxygen consumption rates of C2C12 myotubes transfected with Tomm40 and Tomm22 siRNAs vs. NTC were quantified using the Seahorse 96e Extracellular Flux Analyzer. With the addition of oligomycin, FCCP, and Antimycin A + Rotenone, basal respiration, ATP production, and maximal respiration were quantified. (**D**–**F**) The same experiment carried out in C2C12 cells (**C**,**D**) was conducted in primary hSkMCs. (**G**) Mitochondrial superoxide production with *Tomm40* and *Tomm22* KD in C2C12 cells was quantified by MitoSOX fluorescence probe. All values were normalized to protein concentration by the Bradford assay. All graphical and numeric data represent the mean ± SEM. * *p* < 0.05, ** *p* < 0.01, *** *p* < 0.001, **** *p* < 0.0001 vs. NTC. One-way ANOVA, with Tukey’s post hoc test to identify differences between groups (unless specified, *n* = 4–6 biological replicates).

We next sought to assess the potential role of *TOMM40* and *TOMM22* downregulation by statins in mediating mitochondrial dysfunction by studying the effects of *TOMM40* and *TOMM22* knockdown (KD) in C2C12 and hSkMC myotubes. KD of *Tomm40* and *Tomm22* singly and in combination was performed by a two-step siRNA transfection (Figure 2A), resulting in greater than ~80% knockdown efficiency (Figure 2B). In addition, we compared the KD efficiency in differentiated myotubes vs. undifferentiated myoblasts in C2C12 and hSkMCs and found no differences in *TOMM40* and *TOMM22* mRNA transcript levels (Figure S4). In both the C2C12 and hSkMC myotubes, the basal and maximal oxygen consumption rate (OCR) as well as ATP production were significantly reduced by each condition compared with a non-targeting control (NTC), indicating impaired electron transport chain and mitochondrial function (Figure 2C–F).

To confirm a decrease in mitochondrial respiration due to the suppression of *TOMM40* and *TOMM22* gene expression, we measured mitochondrial superoxide (mitochondrial reactive oxygen species, a.k.a. mitoROS) production using a fluorescence indicator (MitoSOX™) in C2C12 myotubes. This showed increased mitochondrial superoxide production in C2C12 cells transfected with both *Tomm40* and *Tomm22* siRNAs compared with NTC (Figure 2G). Though there was an increased trend observed in cells transfected with *Tomm40* siRNA individually (*p* = 0.171), no significant differences were observed in *Tomm22* KD cells alone. These results demonstrate that KD of either *TOMM40* or *TOMM22* in skeletal myotubes impairs mitochondrial respiration and that their combined KD promotes the generation of mitoROS.

### 2.3. Tomm40 and Tomm22 KD Reduces Cholesterol and CoQ Levels in Mitochondria of C2C12 Myotubes In Vitro

**Figure 3:**
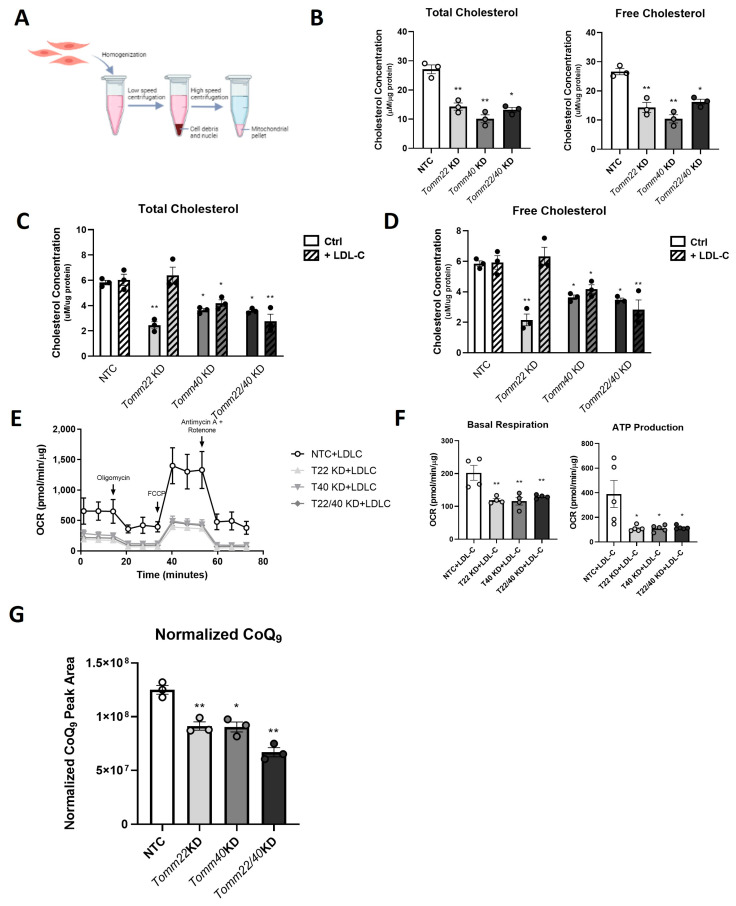
*Tomm40* and *Tomm22* regulate mitochondrial cholesterol content and CoQ levels in C2C12 myotubes. (**A**) Subcellular fractionation was performed to isolate crude mitochondria from whole cells. (**B**) Total and free cholesterol levels in mitochondria isolated from C2C12 myotubes transfected with NTC, *Tomm40*, and *Tomm22* siRNAs, singly and in combination. (**C**,**D**) In a separate experiment, after cells were transfected with siRNAs, 50 µg/mL LDL-C was added to cell media for 24 h. Total and free cholesterol were quantified in the mitochondria using the Amplex Red Cholesterol Assay. (“Ctrl” = control group, “+ LDL-C” = LDL-C treatment group) (**E**) OCR levels were assessed in C2C12 cells after the LDL-C addback, and (**F**) basal respiration and ATP production were quantified using Wave Desktop 2.6 software (*n* = 10–12 biological replicates). (**G**) Total CoQ from isolated mitochondria of NTC, *Tomm40*, *Tomm22*, and *Tomm22/40* KD C2C12 cells were quantified by LC-MS/MS. All values were normalized to protein concentration, measured by BCA. All graphical and numeric data represent the mean ± SEM * *p* < 0.05, ** *p* < 0.01 vs. NTC (without LDL-C addback) by the Welch and Brown–Forsythe ANOVA or one-way ANOVA, with Tukey’s post hoc test to identify differences between groups (unless specified, *n* = 3–6 biological replicates).

Since mitochondria require cholesterol for the maintenance of membrane integrity and proper respiratory function [29], and in light of our recent findings that the KD of *Tomm40* and *Tomm22* reduces the intrahepatic cholesterol content [30], we tested whether suppression of the expression of these genes reduced the mitochondrial cholesterol content in C2C12 myotubes, and whether such an effect contributed to impaired mitochondrial respiration. The isolation of crude mitochondria from C2C12 myotubes was confirmed by the protein expression of Tomm20 and Vdac1 mitochondrial markers on Western blot (Figure S5). Consistent with our hypothesis, a reduction in mitochondrial cholesterol content was observed in both *Tomm40* and *Tomm22* siRNA-transfected cells, singly and in combination, compared with NTC (Figure 3B). To assess whether reduced cholesterol alone was responsible for the disruption of mitochondrial function by *Tomm40* and *Tomm22* KD, we performed a cholesterol addback experiment. The addition of LDL isolated from human plasma rescued the mitochondrial cholesterol content of the *Tomm22* KD but not *Tomm40* KD myotubes (Figure 3C,D). However, cholesterol repletion in the *Tomm22* KD myotubes did not restore reduced mitochondrial ATP production and basal respiration (Figure 3E,F).

Given that CoQ, which shares intermediate biosynthetic steps with cholesterol, plays a central role in the mitochondrial electron transport chain, we next tested the effects of *Tomm40* and *Tomm22* KD on the mitochondrial CoQ levels. Since mice, unlike humans, predominantly synthesize CoQ_9_ (9 prenyl units), we analyzed the CoQ_9_ levels in our murine C2C12 cell model [31]. While *Tomm40* and *Tomm22* KD, singly and in combination, resulted in no differences in the levels of CoQ (Figure S6) in whole cell lysates, there were significant reductions in CoQ_9_ in isolated mitochondria (Figure 3G). Together, these results indicate that TOMM40 and TOMM22 of the TOM complex may impact mitochondrial function in skeletal myotubes at least in part via its effect on the CoQ biosynthesis pathway.

### 2.4. TOMM40 and TOMM22 KD Upregulate Markers of Mitochondrial Fission and Mitophagy in Response to Mitochondrial Damage

**Figure 4:**
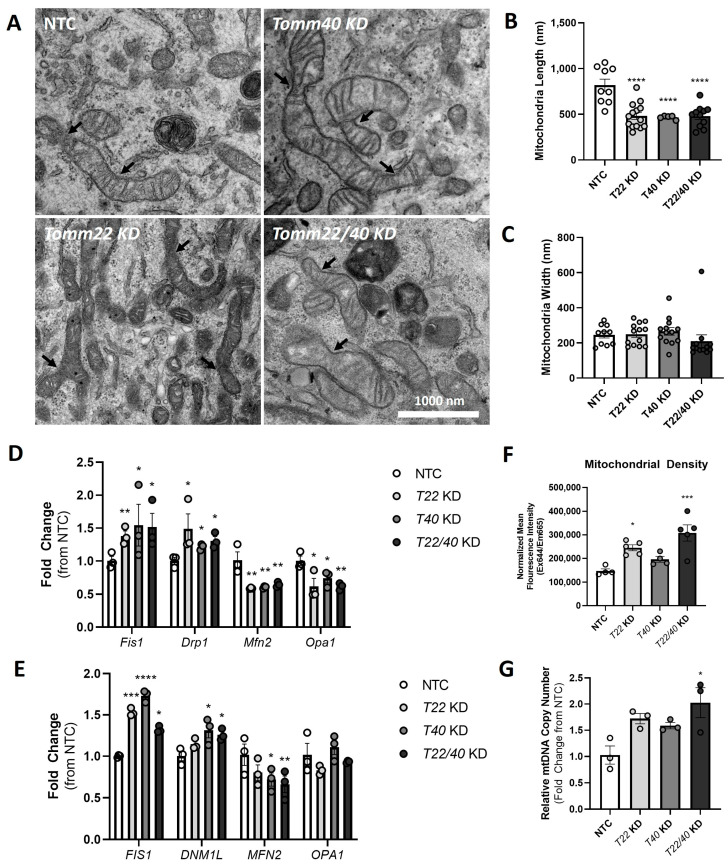
*TOMM40* and *TOMM22* knockdown, singly and in combination, impairs mitochondrial dynamics in skeletal myotubes. (**A**) TEM micrographs of NTC, Tomm40, Tomm22, and Tomm22/40 KD in C2C12 cells. Arrowheads indicate mitochondrial fission events. Analysis of mitochondrial morphology using ImageJ software: (**B**) average mitochondrial length (nm) and (**C**) average mitochondrial width (nm). (*n* = 10–15 cells). (**D**) Mitochondrial fission (*Fis1/FIS1*, *Drp1/DNM1L*) and fusion (*Mfn2/MFN2*, *Opa1/OPA1*) markers were quantified by qPCR in NTC, Tomm22, Tomm40, and Tomm22/40 KD C2C12 cells. (**E**) The experiment was conducted as in (**D**) but with hSkMCs (*n* = 3 biological replicates). (**F**) Mitochondrial density was quantified using a MitoTracker™ Deep Red FM fluorescence probe to measure the average fluorescence intensity and normalized to protein concentration by the Bradford assay (*n* = 10–12 biological replicates). (**G**) Relative mtDNA copy number levels were quantified and normalized to the *B2m* transcript levels (nuclear DNA) by qPCR in siRNA transfected C2C12 cells (*n* = 3 biological replicates). All graphical and numeric data represent the mean ± SEM. * *p* < 0.05, ** *p* < 0.01, *** *p* < 0.001, **** *p* < 0.0001 vs. NTC. One-way ANOVA, with Tukey’s post hoc test to identify differences between groups.

Having observed mitochondrial dysfunction with the suppression of *TOMM40* and *TOMM22*, we next investigated markers of mitochondrial dynamics, a key process that regulates cellular and mitochondrial metabolism [32]. Through the transmission electron microscopy (TEM) of C2C12 myotubes transfected with *Tomm22* and *Tomm40* siRNAs compared with NTC, we observed an increase in constriction events within individual mitochondria, indicative of increased fission events (Figure 4A), along with a significant decrease in average mitochondrial length (Figure 4B), but not width (Figure 4C). This change in mitochondrial morphology led us to hypothesize that *Tomm40* and *Tomm22* KD affects the mitochondrial dynamics in skeletal muscle. Through qPCR in *TOMM22* and *TOMM40* KD C2C12 and hSkMCs, we observed upregulated expression of *FIS1* and *DNM1L/DRP1*, genes that encode markers of mitochondrial fission (Figure 4D,E). This was accompanied by a decrease in the gene expression of the mitochondrial fusion markers *MFN2* and *OPA1*. With a shift toward mitochondrial fission in *TOMM22* and *TOMM40* KD skeletal muscle cells, we observed an increase in mitochondrial density (Figure 4F) and mtDNA copy number (Figure 4G), which together suggest increased mitochondrial damage and support the excessive mitochondrial fragmentation observed.

**Figure 5:**
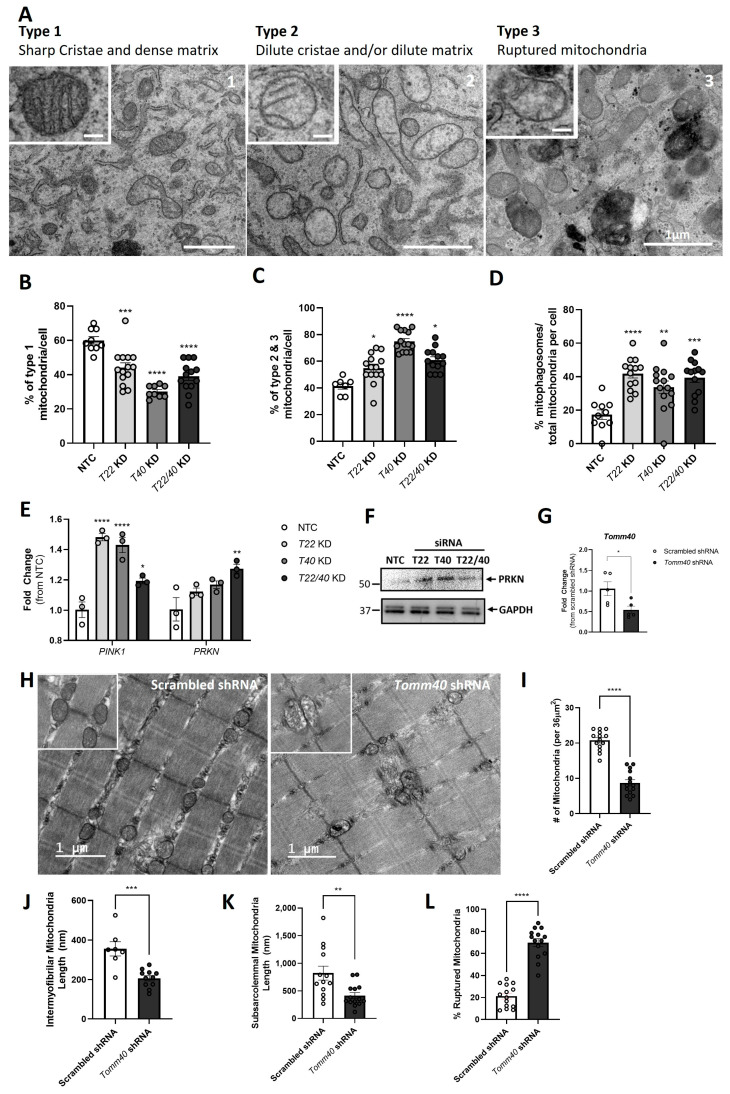
*TOMM40* and *TOMM22* knockdown, singly and in combination, promotes mitochondrial damage and mitophagy in skeletal muscle in vitro and in vivo. (**A**) TEM micrographs of C2C12 myotubes representing types 1 (healthy; A1 image of NTC), 2 (unhealthy; A2 image of *Tomm40* KD), and 3 (damaged/ruptured; A3 image of *Tomm22/40* KD) mitochondria (scale bars = 1 μm; inset scale bars = 200 nm). (**B**) Analysis of mitochondrial morphology and damage in NTC vs. KD C2C12 cells. Bar graph represents percent of cells exhibiting type 1 mitochondria. (**C**) Bar graph represents the percent of types 2 and 3 mitochondria in NTC vs. KD C2C12 cells (for 5B, C, and D: *n* = 10–15 cells, number of mitochondria assessed per cell > 200). (**D**) Percent of mitophagosomes per total number of mitochondria per cell, identified from the TEM images (*n* = 10–15 cells). (**E**) mRNA transcript levels of *PINK1* and *PRKN* (mitophagy) were quantified using qPCR in NTC vs. KD hSkMCs (*n* = 3 biological replicates). (**F**) Representative Western blot of PRKN protein expression in hSkMCs compared with GAPDH control. (**G**) mRNA transcript levels of *Tomm40* (~50% knockdown) were quantified in the gastrocnemius skeletal muscle of male mice injected with AAV8 scrambled vs. *Tomm40* shRNA (*n* = 6/group). (**H**) Representative TEM images of gastrocnemius muscle samples from scrambled vs. *Tomm40* shRNA (*n* = 6/group) male mice. (**I**) Bar graph represents the average number of mitochondria within a surface area of 36 µm^2^, (**J**) length of intermyofibrillar mitochondria (as seen in the TEM images), (**K**) length of sarcolemnal mitochondria, and (**L**) percent of ruptured mitochondria per 36 µm^2^. All graphical and numeric data represent the mean ± SEM. * *p* < 0.05, ** *p* < 0.01, *** *p* < 0.001, **** *p* < 0.0001 vs. NTC or scrambled shRNA by one-way ANOVA, with Tukey’s post hoc test to identify differences between groups.

We further used TEM for analyzing the mitochondrial morphology to assess mitochondrial damage (Figure 5A). KD of *Tomm40* and *Tomm22* resulted in a reduced percentage of type 1 (healthy) mitochondria and an increase in both types 2 and 3 (damaged and ruptured) mitochondria (Figure 5B,C) [33]. Collectively, these results indicate that while there was an increase in new mitochondria created from fission events in knockdown cells (as confirmed in Figure 4F,G), the majority were damaged. Furthermore, we noticed a significant increase in the percentage of mitophagosomes in the *Tomm40* and *Tomm22* KD C2C12 cells (Figure 5D). This observation was confirmed by an increase in the gene and protein expression of the mitophagy markers *PINK1* and *PRKN* in hSkMCs (Figure 5E,F). Accordingly, these results support a compensatory mechanism due to the mitochondrial damage induced by *TOMM40* and *TOMM22* KD in which markers of mitochondrial fission are upregulated, leading to increased mitophagy that removes damaged mitochondria and maintains mitochondrial homeostasis.

In vivo, gastrocnemius skeletal muscle isolated from AAV8-*Tomm40* shRNA injected male mice (14-weeks old) showed a significant reduction in mitochondria number (Figure 5I). In addition, mitochondria morphology was affected so that the lengths of the intermyofibrillar and subsarcolemmal mitochondria were both reduced (Figure 5J,K). Consistent with our in vitro findings, the percent of ruptured mitochondria increased drastically in the *Tomm40* shRNA-induced male mice, indicating signs of mitochondrial damage and dysfunction (Figure 5L) [34]. Gastrocnemius skeletal muscles from AAV8-*Tomm40* shRNA female mice were not affected and presented no indications of mitochondrial damage (Figure S8).

### 2.5. Overexpression of TOMM40 and BCAP31 Rescues Statin-Induced Mitochondrial Dysfunction

**Figure 6:**
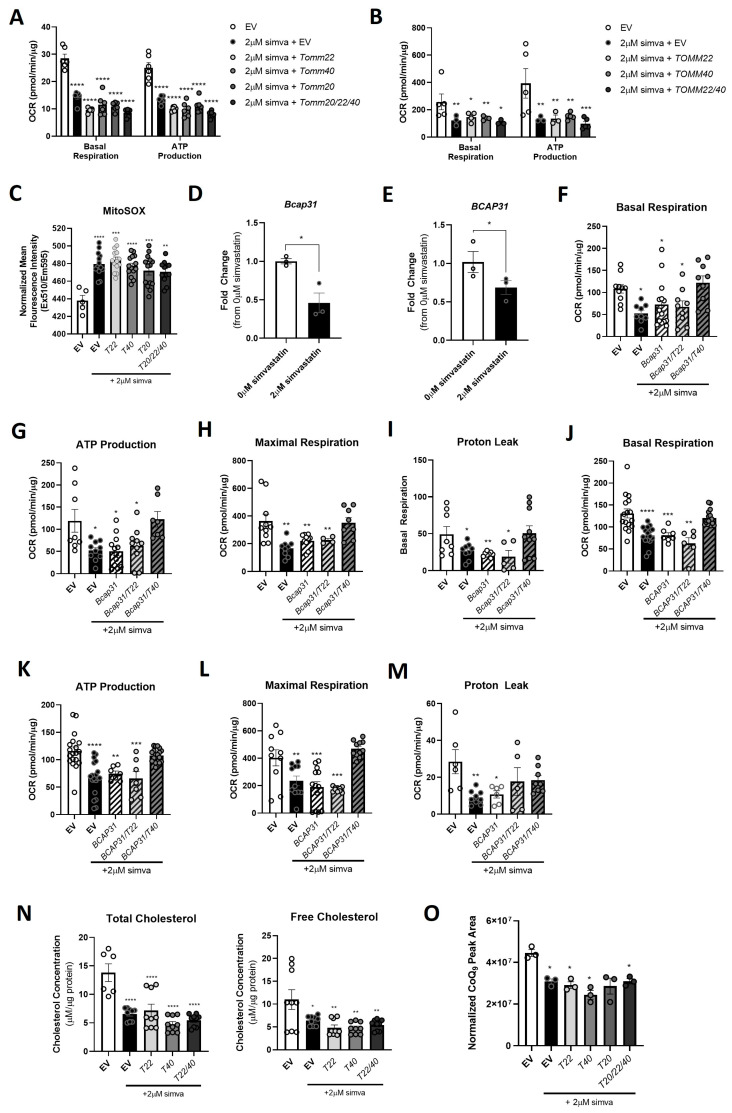
*TOMM40* and *BCAP31* overexpression rescues mitochondrial respiration, ATP production, and proton leak after simvastatin treatment of skeletal muscle cells. (**A**) OCR was measured in C2C12 cells treated with 2 µM simvastatin + empty vector or *Tomm22*, *Tomm40*, *Tomm20* (*T20*), or *Tomm20/22/40* expressing plasmids for 24–48 h. Basal respiration and ATP production determined from the OCR analysis. (**B**) The same experiment was conducted in hSkMCs treated with 2 µM simvastatin + empty vector or *TOMM22*, *TOMM40*, *TOMM22/40* expressing plasmids for 24–48 h (*n* = 10–15 biological replicate). (**C**) mitoROS was determined in C2C12 cells by quantifying the mean mitoSOX™ fluorescence intensity and normalizing to the total protein concentration using the Bradford assay (*n* = 10–12 biological replicates). mRNA transcripts of (**D**) *Bcap31* in C2C12 and (**E**) *BCAP31* in hSkMC myotubes without and with 2 µM simvastatin was quantified by qPCR. (**F**–**I**) Basal respiration, ATP production, maximal respiration, and proton leak were analyzed from OCR recordings in C2C12 cells treated with 2 µM simvastatin + empty vector or *Bcap31*, *Bcap31/Tomm22*, or *Bcap31/Tomm40* expressing plasmids for 24–48 h. (**J**–**M**) Basal respiration, ATP production, maximal respiration, and proton leak were analyzed from OCR recordings in hSkMCs treated with 2 µM simvastatin + empty vector or *BCAP31*, *BCAP31/TOMM22*, or *BCAP31/TOMM40* expressing plasmids for 24–48 h. (**N**) Total and free cholesterol were quantified in the mitochondria of C2C12 myotubes using the Amplex Red Cholesterol Assay (*n* = 3–6 biological replicates). (**O**) Total CoQ_9_ was quantified from mitochondria isolated from C2C12 cells transfected with empty vector (control), 2 µM simvastatin + empty vector, *Tomm22*, *Tomm40*, *Tomm20*, or *Tomm20/22/40* expressing plasmids (*n* = 3 biological replicates). All values were normalized to protein concentration by the BCA. All graphical and numeric data represent the mean ± SEM. * *p* < 0.05, ** *p* < 0.01, *** *p* < 0.001, **** *p* < 0.0001 vs. EV by Welch and Brown–Forsythe or one-way ANOVA, with Tukey’s post hoc test to identify differences between groups.

We next sought to determine whether overexpressing *TOMM40* and *TOMM22*, singly and in combination, could rescue simvastatin-induced effects on mitochondrial function. First, we demonstrated that treatment of C2C12 and hSkMCs with 2 µM simvastatin for 24 h resulted in decreased OCR (both basal and maximal oxygen consumption) and ATP production (Figure 6A,B). Accordingly, mitoROS production was increased by simvastatin in skeletal myotubes (Figure 6E). Introduction of *TOMM40*- and *TOMM22*-containing lentiviral plasmids, singly and in combination, to these simvastatin-treated cells resulted in no changes in the OCR and mitoROS levels (Figure 6A,B). Similarly, over-expressing *Tomm20*, another key component of the TOM complex, did not reverse the simvastatin effect on basal respiration, ATP production, and mitoROS levels in C2C12 cells (Figure 6A–C).

Since TOMM40 is known to impact mitochondrial function by binding to BCAP31 at MERCs [25] and having shown that 2uM simvastatin decreases *BCAP31* expression (Figure 6D,E), we then tested whether TOMM40 and/or TOMM22 required BCAP31 to rescue simvastatin-induced mitochondria dysfunction. We found that overexpressing both *TOMM40* and *BCAP31* in simvastatin-treated C2C12 and hSkMC myotubes resulted in the rescue of ATP production, basal respiration, maximal respiration, and proton leak (Figure 6F–M). However, *TOMM22* in combination with *BCAP31* did not rescue mitochondria function in simvastatin treated cells, consistent with previous evidence of a TOMM40–BCAP31 specific interaction [25].

In accord with the inhibition of mevalonate synthesis by statins^1^, simvastatin (2 µM for 24 h) resulted in reduced levels of two products of the mevalonate/CoQ biosynthesis pathway—cholesterol and CoQ—in mitochondria isolated from C2C12 cells by subcellular fractionation (Figure 6N,O). These effects were not reversed by the overexpression of *Tomm40* and *Tomm22*, or of *Tomm20.* Nor did the overexpression of *Bcap31* with *Tomm40* or *Tomm22* increase the mitochondrial cholesterol levels to the baseline levels (Figure S9)**.** Together, these results indicate that there are other effects of statin besides the downregulation of *Tomm40*, *Tomm22*, *Tomm20*, and *Bcap31*, which are primarily responsible for modulating the cholesterol (Figure S9) and CoQ (Figure 3G) content in the mitochondria of skeletal muscle cells.

### 2.6. TOMM40 and TOMM22 Rescue Statin-Disrupted Regulators of Mitochondrial Fusion and Fission Events

**Figure 7:**
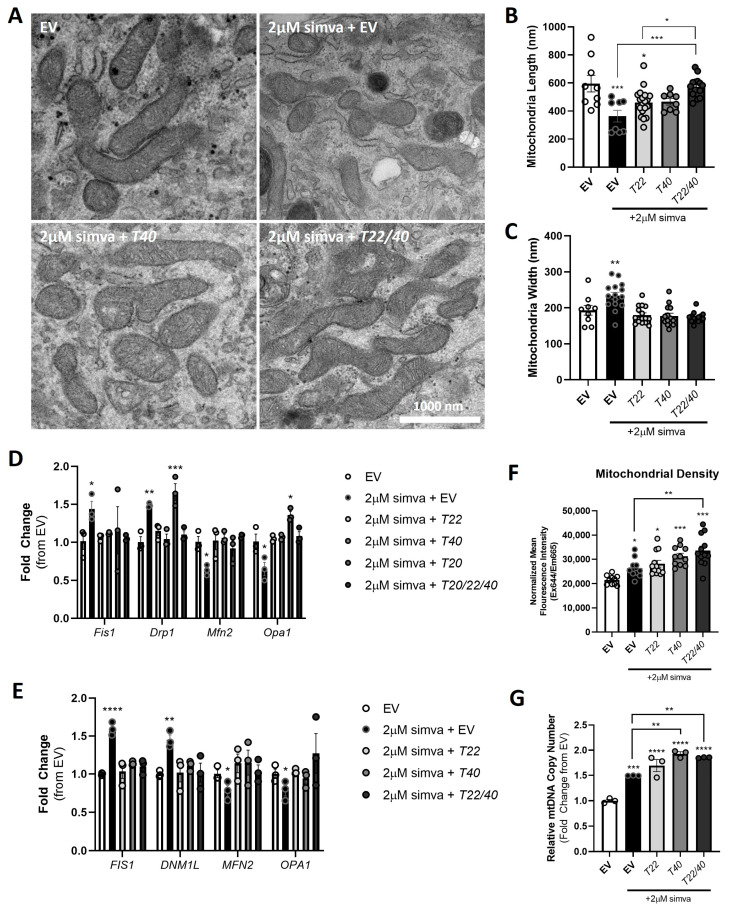
Overexpressing *TOMM40* and *TOMM22*, singly and in combination, suppresses statin-induced mitochondrial fission and promotes fusion in skeletal muscle cells. (**A**) Representative TEM images of mitochondrial morphology in C2C12 cells transfected with empty vector (EV), 2 µM simvastatin + EV, and *Tomm22*, *Tomm40*, or *Tomm22/40* expressing plasmids. (**B**,**C**) Using ImageJ software analysis in conjunction with the TEM images in (**A**), the average mitochondrial length and width (nm) were measured (*n* = 10–15 cells). (**D**) Mitochondrial fission (*Fis1/FIS1*, *Drp1/DNM1L*) and fusion (*Mfn2/MFN2*, *Opa1/OPA1*) markers were quantified by qPCR in EV and 2 µM simvastatin + EV, *Tomm22*, *Tomm40*, *Tomm20*, and *Tomm20/22/40* (*T20/22/40*) overexpressing C2C12 cells. (**E**) The same experiment was conducted as in (**D**) but with hSkMCs (*n* = 3 biological replicates). (**F**) Mitochondrial density was quantified using a MitoTracker™ Deep Red FM fluorescence probe to measure the average fluorescence intensity and was normalized to protein concentration by the Bradford assay (*n* = 10–12 biological replicates). (**G**) Relative mtDNA copy number levels were quantified and normalized to the *B2m* transcript levels (nuclear DNA) by qPCR in siRNA transfected C2C12 cells (*n* = 3 biological replicates). All graphical and numeric data represent the mean ± SEM. * *p* < 0.05, ** *p* < 0.01, *** *p* < 0.001, **** *p* < 0.0001. One-way ANOVA, with Tukey’s post hoc test to identify differences between groups, was performed.

As observed with *Tomm40* and *Tomm22* KD, TEM image analysis of C2C12 myotubes treated with simvastatin 2 µM for 24 h (Figure 7A) resulted in reduced mitochondria length and increased width (Figure 7B,C). The likelihood that this resulted from increased mitochondrial fission events is supported by a qPCR analysis showing that simvastatin treatment resulted in the increased expression of *FIS1* and *DRP1* and reduced expression of *MFN2* and *OPA1* (Figure 7D,E). Furthermore, both mitochondrial density and mtDNA copy number increased in simvastatin-treated C2C12 myotubes compared with the control (Figure 7F,G). Thus, as with *TOMM40* and *TOMM22* KD, simvastatin treatment of skeletal myotubes upregulated markers of mitochondrial fission and damage.

We next tested whether overexpressing *TOMM40* and *TOMM22*, singly and in combination, could rescue these effects of simvastatin on mitochondrial dynamics. Notably, there was a reversal of statin effects on mitochondrial length and width after the addition of *Tomm40* and *Tomm22/40* plasmids in C2C12 myotubes (Figure 7B,C). Moreover, *TOMM40* and *TOMM22* overexpression in both C2C12 and hSkMCs resulted in reversal of the statin effects on the gene expression of the mitochondrial fission and fusion markers described above (Figure 7D,E). These gene expression results were confirmed at the protein level, where simvastatin-induced changes in the protein expression of OPA1 and MFN2 fusion markers, and DRP1 fission markers were restored after overexpressing *TOMM40* or *TOMM22* in hSkMCs (Figure S10). Although *BCAP31* in conjunction with *TOMM40* or *TOMM22* overexpression rescued the gene expression of mitochondrial fission and fusion markers, *BCAP31* alone only rescued *FIS1* gene expression (Figure S11). This suggests that other than BCAP31’s known interaction with FIS1, it is not required for rescuing simvastatin-induced mitochondria dynamics.

**Figure 8:**
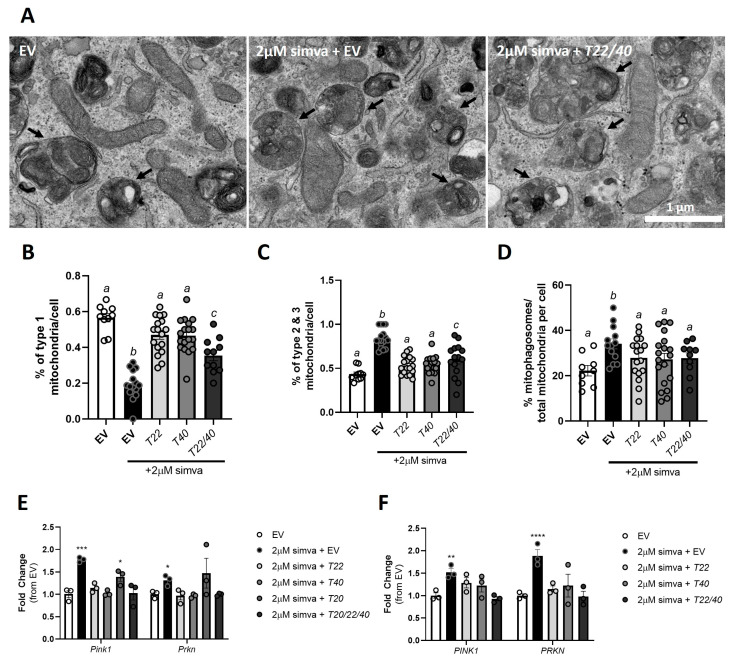
Overexpression of *TOMM40* and *TOMM22*, but not *TOMM20*, rescues simvastatin-induced mitophagy leading to reduced mitochondrial damage. (**A**) TEM micrographs of mitophagosomes found in EV, 2 µM simvastatin + EV, and 2 µM simvastatin + *Tomm22/Tomm40* overexpressing C2C12 cells. Arrowheads indicate mitophagosomes. (**B**) Analysis of mitochondrial morphology and damage in EV or 2 µM simvastatin + EV, *T22*, *T40*, or *T22/40* overexpression in C2C12 cells. Bar graph represents percent of cells exhibiting type 1 mitochondria. (**C**) Bar graph represents percent of types 2 and 3 mitochondria in EV or 2 µM simvastatin + EV, *T22*, *T40*, or *T22/40* overexpression in C2C12 cells (*n* = 10–15 cells). (**D**) Percent of mitophagosomes per total number of mitochondria per cell, identified from TEM images (*n* = 10–15 cells). (**E**,**F**) mRNA transcript levels of *Pink1*/*PINK1* and *Prkn/PRKN* (mitophagy) were quantified using qPCR in EV vs. 2 µM simvastatin + EV, *TOMM22*, *TOMM40*, *TOMM20*, or *TOMM20/22/40* in (**E**) C2C12 and (**F**) hSkMC myotubes (*n* = 3 biological replicates). All graphical and numeric data represent the mean ± SEM. * *p* < 0.05, ** *p* < 0.01, ***p < 0.001, **** *p* < 0.0001 vs. EV by one-way ANOVA, with Tukey’s post hoc test to identify differences between groups. *p* < 0.05 for a vs. b vs. c by two-way ANOVA, with Sidak’s multiple comparisons test.

Interestingly, the addback of *Tomm40* and *Tomm22* plasmids to the simvastatin-treated cells resulted in further increases in mtDNA copy number and mitochondrial density (Figure 7F,G). Together with the evidence that *TOMM40* and *TOMM22* expression reverses statin effects on regulators of mitochondrial dynamics, this suggests that this treatment may suppress statin-induced mitophagy while generating new, healthy mitochondria. Consistent with this hypothesis, we observed by TEM that the simvastatin-treated C2C12 cells had a lower percentage of healthy type 1 mitochondria (sharp cristae and dense matrix) and a higher percentage of type 2 (dilute cristae and/or dilute matrix) and type 3 (ruptured) mitochondria, representing abnormal mitochondrial morphology and signs of mitochondrial injury [7]. In addition, there was an increase in the percentage of mitophagosomes per cell in the simvastatin-treated skeletal muscle (Figure 8D). Consistent with these observations, we demonstrated an increased expression of mitophagy biomarkers *PINK1* and *PARKIN* with simvastatin treatment by qPCR (Figure 8E,F).

We then showed through TEM that overexpressing *Tomm40* and *Tomm22* in statin-treated C2C12 myotubes resulted in an increase in type 1 mitochondria and a reduction in type 2 and type 3 mitochondria, indicating that *Tomm22* and *Tomm40* are able to rescue, at least in part, simvastatin-induced mitophagy. Consistent with this effect, *TOMM22*, *TOMM40*, and *TOMM22/40* (but not Tomm20) overexpression reduced *PINK1* and *PARKIN* expression to the control levels (Figure 8E,F). In summary, these results demonstrate that simvastatin promotes regulators of mitochondrial fission and mitophagy, resulting in an increase in damaged mitochondria, while overexpressing *TOMM22* and *TOMM40* can reverse these effects, thus maintaining mitochondrial quality and homeostasis.

## 3. Discussion

Statin-associated myopathy is the most prevalent adverse effect among statin users, but its mechanism remains unclear [35]. We here report that TOMM40 and TOMM22, key members of the TOM complex whose transcriptional expression is suppressed by simvastatin in skeletal muscle cells, are essential in maintaining mitochondrial function and quality by promoting their oxidative function, retaining CoQ and cholesterol content, and preserving their morphology and dynamics. Consistent with our findings in skeletal muscle, previous studies in epithelial ovarian cancer and HeLa cell lines showed that knockdown of *TOMM40* disrupted mitochondrial membrane potential, ATP, and ROS levels [36,37,38,39]. It has been suggested that mitochondrial dysfunction due to the suppression of *TOMM40* and *TOMM22* is caused by interference with the uptake of mitochondria-targeted proteins [40]. Notably, in the case of *TOMM22*, its suppression or mutation results in the inactivation of mitochondrial proteins due to misfolding in yeast [41] as well as apoptosis of human epithelial and endothelial cells and zebrafish hepatocytes [42].

We also found that *TOMM40* and *TOMM22* KD reduced the cholesterol content in skeletal muscle cells and isolated mitochondria, suggesting that this effect contributes to the mitochondrial dysfunction observed with the KD of these TOM components. Interestingly, adding back LDL cholesterol restored the mitochondrial cholesterol levels in *TOMM22* KD skeletal myotubes, but not in the *TOMM40* KD cells, suggesting that TOMM40 is necessary for maintaining cholesterol levels in muscle mitochondria [43]. However, neither KD group showed an improvement in mitochondrial OCR after the addition of LDL, indicating that under these conditions, mitochondrial respiration and function are regulated by factors other than cholesterol content, or possibly that the added cholesterol was not effectively introduced into the mitochondrial membrane.

CoQ_10_ (humans) or CoQ_9_ (in mice) in the CoQ biosynthesis pathway, essential for regulating the mitochondria electron transport chain and thus mitochondrial function, are dependent on isoprenoids, products of the mevalonate pathway [44]. To date, it is still unclear how CoQ and its precursors are transported across the outer mitochondrial membrane into the mitochondrial matrix of skeletal muscle cells. However, recently, Tai et al. [45] showed in Saccharomyces cerevisiae that isopentenyl pyrophosphate (IPP) molecules, precursors of both CoQ and cholesterol, may enter the mitochondrial matrix via an IPP transporter situated on the inner mitochondrial membrane. Together with our results showing that *TOMM40* and *TOMM22* KD in C2C12 skeletal myotubes resulted in a significant reduction in mitochondrial CoQ content, we suggest that TOMM40 and TOMM22 may affect the transport of proteins required for CoQ biosynthesis, including those involved in the transport of 4-hydroxybenzoate (4-HB) and isoprenoid pyrophosphates, into the mitochondria [46]. Further studies are necessary to determine which transporters and enzymes of the CoQ biosynthesis pathway are recognized and imported into mitochondria by TOMM40 and TOMM22, thus promoting CoQ synthesis [47]. Despite differences in mitochondrial CoQ, the CoQ levels in the whole cell lysates were unaffected, suggesting that decreased mitochondrial CoQ production may be compensated by the promotion of CoQ synthesis in the Golgi apparatus [31,48], a possibility that requires further investigation. Future studies should also investigate the role of CoQ_10_ in hSkMCs to confirm the relevance of these findings in humans.

Mitochondrial dynamics involves the coordination of fusion and fission events that define mitochondria number, size, and morphology [49]. Accumulating evidence has revealed a strong association between mitochondrial function and dynamics [50,51]. In addition, genes regulating mitochondrial dynamics, including *MSTO1*, have been shown to impact other cellular functions including oxidative stress, apoptosis, and mitophagy as well as to induce myopathy and ataxia [52,53,54]. We show here for the first time, using both TEM and expression of mitochondrial dynamic biomarkers, that the KD of TOMM40 and *TOMM22* in skeletal myotubes causes a shift toward increased mitochondrial fission, with a resulting increase in mitophagy as a means of eliminating damaged mitochondria and maintaining mitochondria quality [55,56]. It is plausible that TOMM40 regulates mitochondrial fusion and fission by directly or indirectly interacting with known markers of fission and fusion localized at MERCs such as FIS1, DRP1, and MFN2 [57]. Further studies using fluorescence resonance energy transfer (FRET) with live-cell imaging will be required to assess the interaction and localization of mitochondrial dynamic probes [58].

Additionally, studies in yeast have shown TOMM22 to interact with PINK1, a key regulator of mitochondrial quality and mitophagy [59,60,61,62,63,64]. In mammalian cells, dephosphorylation of TOMM22 impairs PINK1 import into the mitochondria, promoting mitophagy [65]. The effect on mitophagy may be mediated, at least in part, by increased mitoROS as a consequence of mitochondrial dysfunction [66,67]. Consistent with this mechanism, we observed that both mitoROS levels and the mitophagy markers PINK1 and PRKN were upregulated with TOMM40 and TOMM22 KD. Thus, our results indicate TOMM40 and TOMM22 to be key genes in regulating mitochondrial function, dynamics, and mitophagy.

Based on these findings and the evidence that simvastatin downregulates *TOMM40* and *TOMM22* gene expression, we compared the effects of simvastatin exposure with those of *TOMM40* and *TOMM22* knockdown on mitochondrial function and dynamics and tested whether the overexpression of these genes could reverse the statin effects. We showed that simvastatin treatment exerted effects on biomarkers of mitochondrial dynamics and morphology in skeletal muscle cells similar to those observed with both *TOMM40* and *TOMM22* KD. Previous studies in yeast cells reported that statins impair mitochondrial morphology, represented by an increase in aggregated mitochondria, due to disruption of mitochondrial function and membrane potential [68,69,70]. However, we are the first to show that the reduction in mitochondrial length and increased mitochondrial fragmentation in C2C12 cells exposed to simvastatin can be explained by an increase in mitochondrial fission and decrease in fusion events. As with *TOMM40* and *TOMM22* KD, the shift toward mitochondrial fission resulted in increased mitophagy (e.g., via *PINK1* and *PRKN*) to promote the removal of damaged mitochondria [71,72]. We also showed in our in vivo model that the KD of *TOMM40* in mice presented signs of mitochondrial damage in gastrocnemius skeletal muscle, similar to phenotypes observed with statin treatment in previous rodent studies [34,73]. However, these differences were sex-specific, as the *Tomm40* KD had no effect on gastrocnemius skeletal muscle in female mice. This finding is supported by previous in vivo studies that observed female mice showing greater resistance to mitochondrial damage and oxidative stress than males, largely due to the role of estrogen in promoting mitochondrial function and suppressing mitochondrial oxidative stress [74,75]. In addition, since this study used a transient *Tomm40* KD mouse model, future studies employing a stable KD or knockout mouse model, and/or a tissue-specific knockout, will be required to more fully assess the role of Tomm40 in both sexes. Notably, the overexpression of *TOMM40* and *TOMM22* rescued the changes in regulators of mitochondrial dynamics and morphology and mitophagy caused by simvastatin treatment of C2C12 myotubes.

Consistent with previous studies [7,9,10], we showed that simvastatin treatment of C2C12 myotubes resulted in a reduction in ATP production and mitochondrial respiration, together with an increase in mitoROS production. We also observed that simvastatin reduced the free cholesterol content in whole cells as well as in isolated mitochondria [76], an effect that might be linked to impaired mitochondrial structure and membrane potential, rather than mitochondrial function. However, we found that while overexpressing *TOMM40* and *TOMM22* in simvastatin-treated skeletal myotubes rescued mitochondrial structure and morphology, there was no rescue of the intracellular or mitochondrial cholesterol levels, suggesting that reduced cholesterol content is not responsible for the effects of KD on mitochondrial morphology. Moreover, as also expected from statin inhibition of the mevalonate pathway, we showed that simvastatin treated C2C12 cells exhibited a reduction in mitochondrial CoQ levels, an effect that has been suggested to contribute to SAMS [77]. These results paralleled those observed with *TOMM40* and *TOMM22* KD, raising the question as to whether the reduced expression of these genes contributes to these statin effects in muscle, as was the case for the statin-induced changes in mitochondrial morphology and biomarkers of dynamics. In the case of CoQ, the effects of simvastatin and *TOMM40* and *TOMM22* KD were additive (Figure S7). However, overexpression of these genes, including *TOMM20*, another major TOM complex subunit encoding gene [78] as well as *BCAP31*, failed to restore statin impairment of mitochondrial CoQ and cholesterol content. Thus, the reduction in mitochondrial cholesterol and CoQ levels by statin may be due primarily to inhibition of the mevalonate pathway and independent of the reduced expression of genes encoding components of the TOM complex.

TOMM40 has been shown to bind to several ER membrane proteins at MERCs [26]. Among these, BCAP31 has been shown to play a key role in maintaining oxidative phosphorylation, where knockdown of *BCAP31* in U2OS cells showed impaired mitochondrial function and reduced ATP levels [25]. In addition, we have shown that the knockdown of TOMM40 in skeletal muscle cells downregulates *BCAP31* expression (Figure S12). Namba further showed using coimmunoprecipitation that BCAP31 directly binds to TOMM40 and NDUFS4, thereby inducing translocation into the mitochondria of NADH:ubiquinone oxidoreductase (mitochondrial complex I) core subunit 4 (NDUFS4). In addition to TOMM40, BCAP31 is also known to physically interact with FIS1 at MERCs to regulate mitochondrial fusion/fission dynamics and mitochondrial signaling [79,80], Our finding that overexpressing *BCAP31* in simvastatin-treated skeletal myotubes rescues *FIS1* expression is consistent with the possibility that TOMM40 can regulate mitochondria fission through its direct binding to BCAP31, although further studies will be required to confirm whether overexpressing *BCAP31* rescues mitochondrial dynamics and mitophagy in simvastatin-treated skeletal myotubes. We have, however, shown here for the first time that statin exposure of muscle cells reduces *BCAP31* gene expression, and that combined overexpression of *TOMM40* and *BCAP31* in statin-treated C2C12 and hSKMC myotubes is sufficient to rescue impaired mitochondrial oxidative function. This result suggests that statin impairment of mitochondrial respiration can be attributed to disruption of a TOMM40–BCAP31 interaction at MERCs, a mechanism that differs from that responsible for a role of *TOMM40* and *TOMM20* suppression in mediating statin effects on mitochondrial dynamics and structure. Future studies will be required to determine the direct and indirect interactions of TOMM40, TOMM22, and BCAP31 with other proteins at MERCs that may impact adverse mitochondrial effects of statin treatment and predispose to the development of SAMS.

## 4. Materials and Methods

### 4.1. Cell Culture

C2C12 murine myoblasts and primary hSkMCs were purchased from American Type Culture Collection (ATCC, Manassas, VA, USA; CRL-1772 and PCS-950-010). C2C12 cells were cultured in DMEM containing 4.5 g/L glucose and L-glutamine (Gibco, Waltham, MA, USA) supplemented with 10% fetal bovine serum (FBS; Thermo Fisher Scientific, Waltham, MA, USA) and penicillin-streptomycin (Gibco) at 37 °C and 5% CO_2_. After passaging cells, C2C12 myoblasts were differentiated into myotubes by replacing media with DMEM containing 2% horse serum (Gibco). Fresh medium was replaced every 2 days and cells were incubated for 5–7 days to completely differentiate into myotubes before experimentation. For hSkMCs, cells were cultured in mesenchymal stem cell basal medium (PCS-500-030, ATCC, Manassas, VA, USA) supplemented with L-glutamine, 5 ng/mL rh EGF, 10 µM dexamethasone, 5 ng/mL rh FGF-b, 25 µg/mL rh insulin, 4% FBS (PCS-950-040, ATCC, Manassas, VA, USA), and penicillin-streptomycin (Gibco). At 24–96 h after passaging, hSkMCs were differentiated into myotubes using a skeletal muscle differentiation tool (PCS-950-050, ATCC, Manassas, VA, USA) for 2 days before experimentation. Cells were routinely tested for mycoplasma using a MycoAlert™ PLUS Mycoplasma Detection Kit (Lonza, Basel, Switzerland) and only mycoplasma negative cells were used.

### 4.2. siRNA Reverse Transfections

To achieve the knockdown (KD) of *TOMM22* and *TOMM40*, C2C12 and hSkMC skeletal myoblast cells were seeded at 100,000 cells per well in 6-well plates. Upon seeding, cells were reverse transfected with 10 µM of non-targeting control (NTC), *TOMM22* and/or *TOMM40*-targeted siRNAs using Lipofectamine RNAiMax transfection reagent (Thermo Fisher Scientific, Waltham, MA, USA), and Opti-MEM 1 (Gibco, Waltham, MA, USA) for 48 h. All siRNAs were purchased from Thermo Fisher Scientific (Waltham, MA, USA)—human *TOMM22* siRNA (s32549); human *TOMM40* siRNA (s20449); mouse *Tomm22* siRNA (s104588); mouse *Tomm40* siRNA (s79125); Silencer Select Negative Control siRNA (s79125). For C2C12 and hSkMC, cells were transfected twice, at day 0 (myoblasts) and days 2 (hSKMC) or 3 (C2C12; myotubes) in differentiation media and then harvested after day 5 for experimentation.

### 4.3. Overexpression Plasmids

Human and mouse pCMV-EGFP expressing-*TOMM40/Tomm40* (human: NM_001128916.2, mouse: NM_016871.2), *TOMM22/Tomm22* (human: NM_020243.5, mouse: NM_172609.3), *Tomm20* (mouse: NM_024214.2), *BCAP31/Bcap31* (human: NM_001139457.2, mouse: NM_012060.5), and empty vector (EV; ORF_stuffer) plasmids stored in bacterial glycerol stocks were purchased from VectorBuilder Inc. (Chicago, IL, USA). Expression plasmids were cultured on Luria-Bertani (LB) Agar plates containing ampicillin at 37 °C. Single colonies were selected and grown separately in LB broth at 37 °C with continuous shaking (225 rpm) overnight. DNA plasmids were then purified and extracted using the ZymoPURE II Plasmid Midiprep Kit (Zymogen, Irvine, CA, USA) according to the manufacturer’s protocol. For overexpression studies, both C2C12 and hSkMCs were first differentiated into myotubes using the respective differentiation media. After differentiation, cells were transiently transfected with purified *TOMM40/Tomm40*, *TOMM22/Tomm22*, and *Tomm20* expression plasmids, singly and in combination, or matched empty vector, using the Lipofectamine 3000 transfection reagent (Thermo Fisher Scientific, Waltham, MA, USA). In parallel, 2 µM simvastatin was added to the cell media, and cells were collected after 24–48 h. Simvastatin was obtained as a gift from Merck Inc. (Whitehouse Station, NJ, USA) and activated to the β-hydroyxyacid form prior to use as previously described [81].

### 4.4. Animal Studies

The 6-week old C57BL/6J male and female mice (*n* = 6 per group) were purchased from Jackson Laboratory (Bar Harbor, ME, USA) and placed on a high-fat Western diet. At 8 weeks of age, mice were intraperitoneally (IP) injected with either 4 × 10^11^ GC AAV8-*Tomm40* shRNA or AAV8-CMV-null as a control (VectorBuilder, Chicago, USA). Weekly bodyweight and food intake measurements were recorded. At 14-weeks old, mice were terminated and gastrocnemius muscle tissues were collected and immediately fixed in 2% glutaraldehyde +2% paraformaldehyde solution for transmission electron microscopy.

### 4.5. Mitochondrial Respiration Measurements

In vitro oxygen consumption rate was measured in fully differentiated C2C12 and hSkMC skeletal myotubes with an Agilent Seahorse XFe96 Extracellular Flux Analyzer (Santa Clara, CA, USA). Skeletal myoblast cells were seeded at 1000 per well in 96-well plates and were incubated for 5–7 days until the myoblasts were fully differentiated into myotubes with a cell density of ~30,000–40,000 cells per well (3–4 × 10^5^ cells/mL), measured with the Countess 3™ automated cell counter (Invitrogen, Carlsbad, CA, USA). Prior to the start of the experiment, skeletal myotubes were incubated in XF assay medium (Agilent, Santa Clara, CA, USA) supplemented with 2 mM sodium pyruvate (Gibco, Waltham, MA, USA), 2 mM GlutaMAX™ (Gibco, Waltham, MA, USA), and 10 mM glucose (Sigma, St. Louis, MO, USA) at pH 7.4. During experimentation, 1.5 µM oligomycin, 2 µM FCCP, and 2 µM Antimycin A + Rotenone (Seahorse XF Cell Mito Stress Test Kit, Agilent, Santa Clara, CA, USA) was added sequentially via injection ports to calculate the basal and maximum respiration and ATP production. Oxygen consumption rate (OCR) values were presented with non-mitochondrial oxygen consumption deducted and normalized to total protein concentration per well using the Bradford assay.

### 4.6. Fluorescence Quantification

To detect mitochondria superoxide production in C2C12 cells, myotubes were incubated with 5 µM MitoSOX™ Red (Molecular Probes, Eugene, OR, USA) for 20 min at 37 °C. Cells were then rinsed twice in pre-warmed 1× phosphate-buffered saline (PBS), which was then replaced with phenol red-free DMEM (21063029; Gibco, Waltham, MA, USA) supplemented with glucose and sodium pyruvate. Fluorescence was detected and quantified at an excitation/emission of 510/595 nm (with an Ex/Em bandwidth of 10 nm) using an Agilent BioTek™ microplate fluorescence spectroscopy reader (Santa Clara, USA) and BD LSRFortessa™ Cell Analyzer flow cytometer (Franklin Lakes, USA) following the protocol of Kauffman et al. [82]. To minimize the effects of uneven cell distribution, we employed orbital averaging during the readings. To quantify mitochondrial density, MitoTracker™ Deep Red FM (100 nM) was added to the C2C12 cells for 30 min at 37 °C, washed twice with 1× PBS, and replaced with phenol red-free DMEM supplemented with glucose and sodium pyruvate (Gibco, Waltham, MA, USA). MitoTracker fluorescence was quantified at an excitation/emission of 644/665 nm. All absorbance readings were normalized to total protein concentration by the Bradford assay.

### 4.7. Isolation of Mitochondria by Subcellular Fractionation

Mitochondria were isolated from C2C12 and hSkMCs according to the method of Wettmarshausen and Perocchi [83]. Cells were rinsed in 1× PBS twice, dislodged with 0.25% Trypsin-EDTA (Gibco), washed again in 1× PBS, and centrifuged at 600× *g* for 5 min at 4 °C. Pelleted cells were resuspended in MSHE + BSA buffer (210 mM mannitol, 70 mM sucrose, 5 mM HEPES, 1 mM EGTA, and 0.5% BSA, at 7.2 pH). Samples were transferred to a small glass dounce and homogenized. The homogenate was centrifuged at 600× *g* for 10 min at 4 °C, and the supernatant was extracted and centrifuged at 8000× *g* for 10 min at 4 °C. The isolated pellet containing the purified crude mitochondria was dried down by nitrogen gas and snap frozen in liquid nitrogen for quantification of the mitochondrial cholesterol and CoQ levels.

### 4.8. Lipid Extraction for LC-MS/MS

C2C12 whole cell lysates and isolated mitochondria pellets were resuspended in 100 µL of 150 mM KCl. Ten percent of the cell suspension was removed from each tube and placed into a new tube. The extra 10% was later used in a bicinchoninic acid (BCA) assay to measure the relative protein content in each sample. Protein content derived from the BCA assay was used to normalize the CoQ measurements.

The remaining 90% of the cell suspension was mixed with glass beads and 600 µL of cold methanol containing 0.25 µM CoQ6 (CoQ6 was used to normalize for the total CoQ extracted). Cell suspensions were subjected to lysis on a vortex genie at 4 °C for 10 min. Afterward, 400 μL of cold petroleum ether was added to each tube, and vortexing was repeated for 3 min. To separate the petroleum ether and methanol phases, the tubes were centrifuged at 1100× *g* for 3 min, and the top (petroleum ether) phase was collected into a new tube (Tube B). Again, 400 µL of petroleum ether was added to each tube containing methanol, and the vortexing/centrifuge steps were repeated. The final top layer was collected and added to Tube B. Petroleum ether was dried under a stream of argon gas, and dried lipids were resuspended in 50 μL of mobile phase (78:20:2 methanol:isopropanol:ammonium acetate).

### 4.9. Measurement of CoQ_9_ by LC-MS/MS Lipidomics

LC-MS/MS Lipidomics Data Acquisition: A Vanquish Horizon UHPLC system (Thermo Scientific, Waltham, MA, USA) connected to an Exploris 240 Orbitrap mass spectrometer (Thermo Scientific, Waltham, MA, USA) was used for targeted LC-MS analysis. A Waters Acquity CSH C18 column (100 mm × 2.1 mm, 1.7 μm) was held at 35 °C with the flow rate of 0.3 mL/min for lipid separation. A Vanquish binary pump system was employed to deliver mobile phase A consisting of 5 mM ammonium acetate in ACN/H2O558 (70/30, *v*/*v*) containing 125 μL/L acetic acid, and mobile phase B consisting of 5 mM ammonium acetate in IPA/ACN (90/10, *v*/*v*) containing 125 μL/L acetic acid. The gradient was set as follows: B was at 2% for 2 min and increased to 30% over the next 3 min, then further ramped up to 50% within 1 min and to 85% over the next 14 min, and then raised to 99% over 1 min and held for 4 min before being re-equilibrated for 5 min at 2% B. Samples were ionized by a heated ESI source with a vaporizer temperature of 350 °C. Sheath gas was set to 50 units, auxiliary gas was set to 8 units, sweep gas was set to 1 unit. The ion transfer tube temperature was kept at 325 °C with a 70% RF lens. Spray voltage was set to 3500 V for positive mode. The targeted acquisition was performed with tMS2 (targeted MS2) mode: tMS2 mode was for measuring CoQ_6_ (*m*/*z* 591.4408, internal standard) and CoQ_9_ (*m*/*z* 795.6286) in positive polarity at the resolution of 15,000, isolation window of 2 *m*/*z*, normalized HCD collision energy of either 40% or stepped HCD energies of 30% and 50%, with a standard AGC target and auto maximum ion injection time.

Data Analysis: Targeted quantitative analysis of all acquired compounds was processed using TraceFinder 5.1 (Thermo Scientific, Waltham, MA, USA) with the mass accuracy of 5 ppm. The result of peak integration was manually examined.

### 4.10. Lipid Extraction and Intracellular Cholesterol Quantification

Cholesterol was extracted from cells with hexane–isopropanol (3:2, *v*/*v*), dried under nitrogen gas and reconstituted with buffer (0.5 M potassium phosphate, pH 7.4, 0.25 M NaCl, 25 mM cholic acid, 0.5% Triton X-100). Intracellular cholesterol levels were then quantified with the Amplex Red Cholesterol Assay Kit (Life Technologies, Carlsbad, CA, USA) according to the manufacturer’s protocol.

### 4.11. Sample Preparation for Electron Microscopy

C2C12 cells were grown on MatTek glass bottom dishes (P35G-1.5-14-C, MatTek, Ashland, OR, USA) and fixed in 2% glutaraldehyde + 2% paraformaldehyde solution (prepared by Electron Microscopy Lab, Berkeley, CA, USA) for 24 h. After fixation, cells and tissues were washed 3-times for 5 min in 0.1 M sodium cacodylate buffer, pH 7.4. Samples were then post-fixed in 1% osmium tetroxide + 1.6% potassium ferricyanide (KFECn) in 0.1 M sodium cacodylate buffer for 30 min, before undergoing three washes at 15 min each. Cells were then dehydrated in a serial diluted ethanol solution of 30, 50, 70, 90, and 100% for 10 min each. Samples were infiltrated with 50% Epon-Araldite resin (containing benzyldimethylamine (BDMA) accelerator), followed by 100% resin for 1 h each. Excess resin was removed from the MatTek dishes containing cells and polymerized at 60 °C for 48 h.

### 4.12. Transmission Electron Microscopy

Using a dissecting blade, cells embedded in resin were removed from MatTek dishes and mounted on resin-embedded blocks for sectioning. Serial sections of 70–150 nm thickness were cut on a Reichert-Jung Ultracut E microtome and set on 1 × 2-mm slot grids covered with 0.6% Formvar film. Sections were then post-stained with 1% aqueous uranyl acetate for 7 min and lead citrate for 4 min [84]. Images of cell samples were taken on an FEI Tecnai 12 transmission electron microscope (Hillsboro, CA, USA) equipped with a 2k × 2k CCD camera with a 40 Megapixel/s readout mode. Images were analyzed using ImageJ (version 1.54p) software according to the method by Lam et al. [85]. Quantification of mitochondrial morphology and mitophagy were performed double-blinded where the groups and images were randomized. Types of mitochondria were evaluated by trained staff at the UC Berkeley electron microscopy laboratory using the method described by Shults et al. [86].

### 4.13. Immunoblotting

Cells were washed with PBS and lysed in M Cellytic Lysis Buffer containing 1% protease inhibitor (Halt ™ Protease Inhibitor Cocktail; Thermo Scientific, Waltham, MA, USA) for 15 min with gentle vortexing. The cell lysate was centrifuged at 14,000× *g* for 15 min, the supernatant was collected, and the protein concentration was measured by the Bradford assay. To ensure balanced loading across conditions, the total protein concentration in the control and treatment samples were normalized to achieve an equal protein loading per well. Proteins were separated on a 4–20% Tris-polyacrylamide gradient gel (Bio-Rad, Hercules, CA, USA) and transferred onto a nitrocellulose membrane using the iBlot™ 2 Gel Transfer Device (Thermo Fisher Scientific, Waltham, MA, USA). Membranes were blocked in Tris-buffered saline with 0.1% Tween (TBST) + 5% milk for 2 h to minimize non-specific antibody binding. Membranes were then incubated with primary antibodies: TOMM40 (E6Q3Z), TOMM22 (E3F4M), TOMM20 (D8T4N), VDAC1 (D73D12), MFN2 (D1E9), OPA1 (D6U6N), DRP1 (D6C7), GAPDH (14C10) rabbit monoclonal antibodies, and PARKIN (Prk8) mouse monoclonal antibody (Cell Signal) diluted 1:1000 (*v*/*v*) in TBST overnight on a rotating platform at 4 °C. After washing in TBST, membranes were incubated with secondary antibodies, anti-rabbit IgG (7074) and anti-mouse IgG (7076), HRP-linked antibodies (Cell Signal, Danvers, MA, USA) at 1:2500 (*v*/*v*) dilution, for 30 min before a last series of washes. SuperSignal™ West Pico PLUS Chemiluminescent Substrate (Thermo Fisher Scientific, Waltham, MA, USA) was added to the membrane to visualize proteins [87].

### 4.14. RT-qPCR and mtDNA Copy Number

RNA was extracted from cells using the RNeasy Mini Qiacube Kit with the Qiacube Connect (Qiagen, Venlo, The Netherlands) according to the manufacturer’s protocol. cDNA synthesis from total RNA was performed using High-Capacity cDNA Reverse Transcription Kits (Applied Biosystems, Waltham, MA, USA). Primers obtained from Elim Biopharmaceuticals were run with SYBR™ Green qPCR Master Mix (Thermo Fisher Scientific, Waltham, MA, USA) on an ABI PRISM 7900 Sequence Detection System to quantify the mRNA transcript levels. RT-qPCR primers used in this study are listed in Table S1. The mean value of triplicates for each sample was normalized to GAPDH as the housekeeping gene. The delta delta Ct method was used to calculate 2-fold change in mRNA expression compared with the control group.

Total DNA was isolated from C2C12 cells using the DNeasy Blood and Tissue Kit (Qiagen, Venlo, The Netherlands). qPCR was performed with SYBR™ Green qPCR Master Mix (Thermo Fisher Scientific, Waltham, MA, USA) on an ABI PRISM 7900 Sequence Detection System according to the protocol outlined by Quiros et al. [30]. Primers for mouse mtDNA (mMitoF1: 5′-CTAGAAACCCCGAAACCAAA-3′, mMitoR1: 5′-CCAGCTATCACCAAGCTCGT-3′) and mouse B2M (mB2MF1: 5′-ATGGGAAGCCGAACATACTG-3′, mB2MR1: 5′CAGTCTCAGTGGGGGTGAAT-3′) were used to amplify mtDNA and nuclear DNA, respectively. mtDNA copy number was determined by normalizing mtDNA to nuclear DNA. The delta delta Ct method was used to calculate fold change in mtDNA copy number.

### 4.15. Statistical Analysis

All data are presented as the mean ± standard error of the mean (SEM). *N*-values in the figures refer to biological replicates, and at least three replicates were conducted per condition and experiment. *p*-values were calculated using Student’s *t*-tests for two groups. To compare more than two groups, one-way analysis of variance (ANOVA) or Welch and Brow–Forsythe ANOVA with Tukey’s post hoc test were used. Analyses were performed using GraphPad Prism 9 software (GraphPad Software, Inc., San Diego, USA). *p < 0.05* was considered statistically significant.

## 5. Conclusions

This study demonstrates that TOMM40 and TOMM22, major members of the TOM complex, have key roles in maintaining mitochondrial function, composition, and dynamics in skeletal muscle cells. We further highlight that downregulation of these genes by simvastatin treatment is in part responsible for the mitochondrial phenotypes induced in these skeletal muscle cells, in which the overexpression of *TOMM40* and *TOMM22* restored markers of mitochondrial dynamics and morphology. Furthermore, the overexpression of both *TOMM40* and *BCAP31* was required to restore simvastatin-induced mitochondrial dysfunction in vitro. This study also demonstrated reductions in mitochondrial cholesterol and CoQ_10_ content with statin exposure of skeletal muscle cells but that these effects were independent of changes in the expression of genes of the TOM complex. We conclude that, since mitochondrial dysfunction is known to have a key role in mediating SAMS, our findings suggest a plausible mechanism for the contribution of statin-induced suppression of *TOMM40* and *TOMM22* to their pathogenesis.

## Data Availability

The original contributions presented in this study are included in the article/Supplementary Materials. Further inquiries can be directed to the corresponding author.

## Supplementary Materials

The following supporting information can be downloaded at: https://www.mdpi.com/article/10.3390/ijms262210977/s1.

## Abbreviations

The following abbreviations are used in this manuscript:

TOM	Translocase of outer mitochondrial membrane
SAMS	Statin-associated muscle symptoms
hSkMCs	Primary human skeletal muscle cells
KD	Knockdown
MERCs	Mitochondria-ER contact sites
CoQ	Coenzyme Q
OCR	Oxygen consumption rate
mitoROS	Mitochondrial reactive oxygen species
TEM	Transmission electron microscopy
