# Supplementary material for: Targeting TOMM40 and TOMM22 to Rescue Statin-Impaired Mitochondrial Function, Dynamics, and Mitophagy in Skeletal Myotubes
Source: Int J Mol Sci. 2025 Nov 13;26(22):10977. doi: 10.3390/ijms262210977 (PMC12652557; doi:10.3390/ijms262210977)
Supplement: Supplementary file 1 [file ijms-26-10977-s001.zip › ijms-3963968-supplementary.pdf]

SUPPLEMENTAL MATERIAL:

Mouse Primers					Human Primers			
Gene	Direction	Primer sequence	Experiment		Gene	Direction	Primer sequence	Experiment
Tomm40	Forward	GAA GAT GGG AGC TGC GGA T	Fig 1		*TOMM40*	Forward	AGG AGG GCA CTG TCA TGT CT	Fig 1
Tomm40	Reverse	AAG TGG TAG TTG GAC TCC CC	Fig 1		*TOMM40*	Reverse	TGG TCA CTG GCT TTG TGG TA	Fig 1
Tomm22	Forward	CAG CTT TGT GGA TTG GGA CC	Fig 1		*TOMM22*	Forward	AAT GGA GCA ACA GCA GCA AC	Fig 1
Tomm22	Reverse	GGC CCT AAA AGT ATC TGC CG	Fig 1		*TOMM22*	Reverse	CAG GAA GTG AGG GTA GAG CC	Fig 1
Tomm20	Forward	CCC CAA CTT CAA GAA CAG GC	Fig 1		*TOMM20*	Forward	CAG CTT GGT GAA GAG TTA CTA GC	Fig 1
Tomm20	Reverse	GTC GGA AGC TTG GTC AGA AG	Fig 1		*TOMM20*	Reverse	GGC GTA GAC CAT CTG ACA AA	Fig 1
Tomm70a	Forward	GAC AAG GAG GGA GAG GCT TT	Fig 1		*TOMM70*	Forward	GCC AGG CAT ATA CGG GAA AC	Fig 1
Tomm70a	Reverse	AGT AGG TAG AAG GTG GCT CG	Fig 1		*TOMM70*	Reverse	GCG TAT AGT GCA TAG CCT TCG	Fig 1
Tomm5	Forward	GGA GGA GAT GAA GCG GAA GA	Fig 1		*TOMM5*	Forward	TTC TCA TCT ACG TGG CCC TC	Fig 1
Tomm5	Reverse	TCA CCA GGT CTT CAT GCC AT	Fig 1		*TOMM5*	Reverse	CGT TCA GCT CAG TTC GAA GG	Fig 1
Tomm6	Forward	TCG GTT GGT ACG TGC TTA GT	Fig 1		*TOMM6*	Forward	CAA CGT GGG AGA TTG GCT TC	Fig 1
Tomm6	Reverse	CGC AGC AAA GAG TCC CAA AT	Fig 1		*TOMM6*	Reverse	CGC AGC AAA GAG TCC CAA AT	Fig 1
Tomm7	Forward	CCA TGG TGA AGC TGA GCA AA	Fig 1		*TOMM7*	Forward	GGT GAA GCT GAG CAA AGA GG	Fig 1
Tomm7	Reverse	CAG GAT CTG CAC CCC TTG TA	Fig 1		*TOMM7*	Reverse	CCC AGG TAA ATC ACA AGA GGG	Fig 1
Ldlr	Forward	CGC GGA TCT GAT GCG TCG CT	Fig S1		*FIS1*	Forward	AGG AGG AAC AGC GGG ATT AC	Fig 4
Ldlr	Reverse	CGG CCC TGG CAG TTC TCT GG	Fig S1		*FIS1*	Reverse	GTT CCT TGG CCT GGT TGT TC	Fig 4
Hmgcr	Forward	CTT TCA GAA ACG AAC TGT AGC TCA C	Fig S1		*DNM1L*	Forward	GCA ACT GGA GAG GAA TGC TG	Fig 4
Hmgcr	Reverse	CTA GTG GAA GAT GAA TGG ACA TGA T	Fig S1		*DNM1L*	Reverse	GCA CAT CTA GCA GGT TCA CG	Fig 4
Fis1	Forward	AGA GGA ACA GCG GGA CTA TG	Fig 4, 8		*MFN2*	Forward	GCA CTT TGT CAC TGC CAA GA	Fig 4
Fis1	Reverse	CTC CTT GGC CTG GTT GTT CT	Fig 4, 8		*MFN2*	Reverse	CAC TTT CAT GTG CCT CCG AG	Fig 4
Drp1	Forward	TCT CAA GGT TTT CTC GCC CA	Fig 4, 8		*OPA1*	Forward	TAC TAG GAT CGG CTG TTG GG	Fig 4
Drp1	Reverse	TGA CGG CGA GGA TAA TGG AA	Fig 4, 8		*OPA1*	Reverse	TTT CCC ACA CAA TGT CAG GC	Fig 4
Mfn2	Forward	CTC ACA GAG GGC TCA GAA GA	Fig 4, 8		*PINK1*	Forward	TCA ATC CCT TCT ACG GCC AG	Fig 5
Mfn2	Reverse	CCA CAT CAC ACT CAC CAT GC	Fig 4, 8		*PINK1*	Reverse	CCA ACT GTC TCA CGT CTG GA	Fig 5
Opa1	Forward	GAC TCT CAG TGA AGG CCC TC	Fig 4, 8		*PRKN*	Forward	TCA GGT TCA ACT CCA GCC AT	Fig 5
Opa1	Reverse	CGG GGC TAA CAG TAC AAC CT	Fig 4, 8		*PRKN*	Reverse	TGC ACA GTC CAG TCA TTC CT	Fig 5
Pink1	Forward	ATC TTT GGG CTT GCC AAT CC	Fig 9		*BCAP31*	Forward	GAC TCT CAT TTC GCA GCA GG	Fig 6, S8
Pink1	Reverse	CTA GAA GAT GCT CGC CCC AG	Fig 9		*BCAP31*	Reverse	GAG CTG GTC ATT CTC CTC CA	Fig 6, S8
Prkn	Forward	ACC CAC CTA CAA CAG CTT TT	Fig 9					

Prkn	Reverse	ACT GAA CTC GGA GCT TTC CA	Fig 9					
Bcap31	Forward	GCC TGA AGA ATG ACC TGA GG	Fig 6					
Bcap31	Reverse	TAA GGC CCT CAG ACT GCT TC	Fig 6					

Table S1. RT-qPCR primers employed in this study.

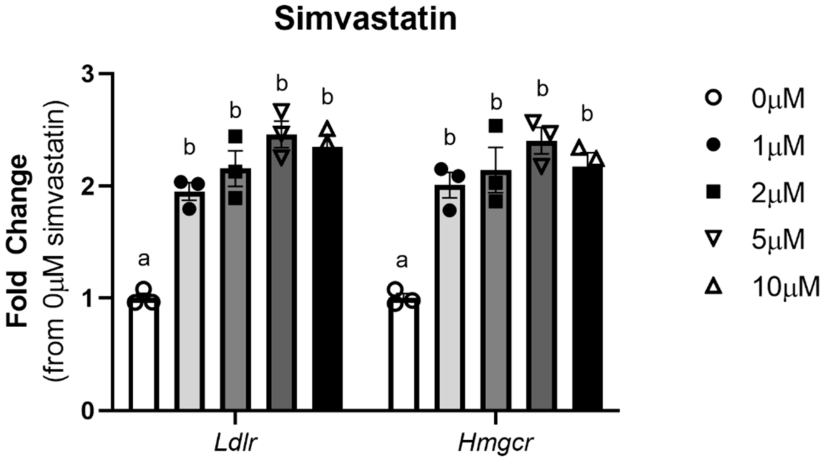

Figure S1. Simvastatin treatment of C2C12 myotubes upregulates *Ldlr* and *Hmgcr* mRNA transcripts in a dose-dependent manner. Differentiated C2C12 myotubes were treated with 0, 1, 2, 5 or 10 µM simvastatin for 48 hrs. All numeric data represent mean ± SEM. **p<0.05*, ***p<0.01*, ****p<0.001*, *****p<0.0001* vs. 0 µM simvastatin by one-way ANOVA, with Tukey's post-hoc test to identify differences between groups. (*n = 3* biological replicates)

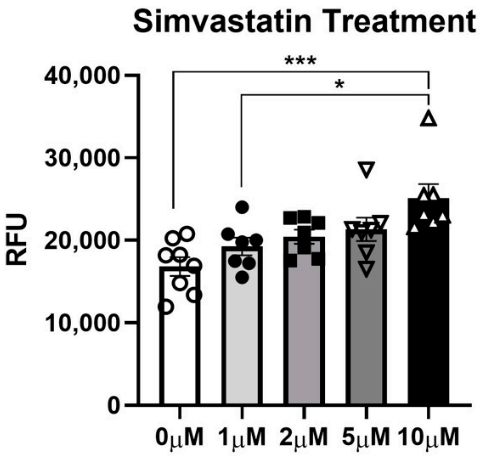

Figure S2. Dose-dependent cell apoptosis identified in C2C12 myotubes. Differentiated C2C12 myotubes were treated with 0, 1, 2, 5 or 10 µM simvastatin for 48 hrs and apoptosis was assessed using an EarlyTox Caspase-3/7 colorimetric detection kit with a spectrophotometer. RFU = relative fluorescence units. All numeric data represent mean ± SEM. *$p<0.05$, ***$p<0.001$ vs. 0 µM simvastatin by one-way ANOVA, with Tukey's post-hoc test to identify differences between groups. (n = *6-12* biological replicates)

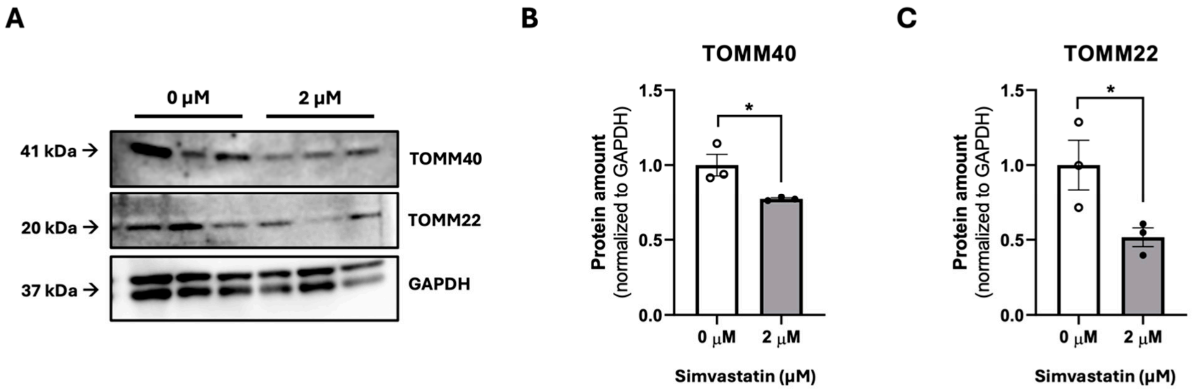

Figure S3. Simvastatin treatment of primary hSkMC myotubes decreases TOMM40 and TOMM22 protein expression. Differentiated primary hSkMC myotubes were treated with 2 µM simvastatin for 24 hrs. (A) Representative western blot of TOMM40 and TOMM22 protein expression of 0 µM vs. 2 µM simvastatin treatment in primary hSkMC myotubes. GAPDH was used as the control. Relative protein amount of (B) TOMM40 and (C) TOMM22 protein expression in 0 vs. 2 µM simvastatin treatment in primary hSkMC myotubes. All numeric data represent mean ± SEM. *$p<0.05$ vs. 0 µM simvastatin by Student's t-test. ($n = 3$ biological replicates)

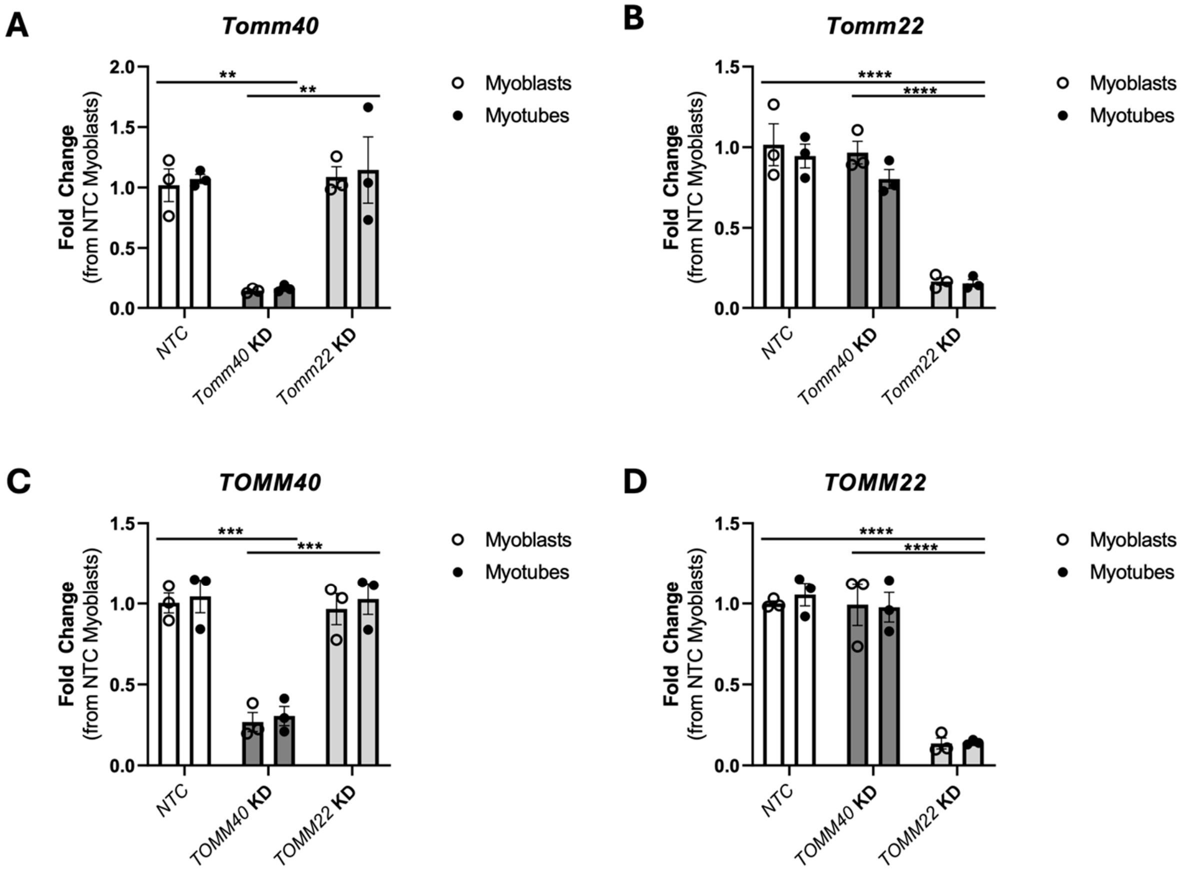

Figure S4. *TOMM40* and *TOMM22* knockdown efficiency in skeletal myoblasts vs. myotubes. Undifferentiated skeletal myoblasts and differentiated skeletal myotubes were transfected with NTC, *TOMM40,* or *TOMM22* siRNAs for 48 hrs. mRNA transcripts show no differences in (A) *Tomm40* and (B) *Tomm22* between undifferentiated vs. differentiated C2C12 cells by qPCR. mRNA transcripts show no differences in (C) *TOMM40* and (D) *TOMM22* between undifferentiated vs. differentiated hSkMC cells by qPCR. All numeric data represent mean ± SEM. $^{**}p<0.01$, $^{***}p<0.001$, $^{****}p<0.0001$ vs. NTC by one-way ANOVA, with Tukey's post-hoc test to identify differences between groups. ($n = 3$ biological replicates)

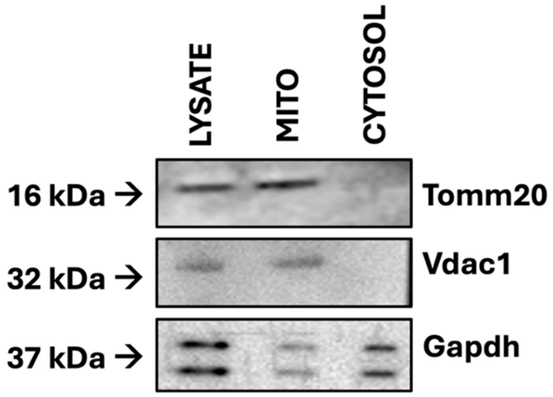

Figure S5. Fractionation of cytoplasmic and mitochondrial proteins prepared from C2C12 skeletal myotubes. Representative western blot of Tomm20 and Vdac1 (mitochondrial markers), and Gapdh (control) protein expression in lysate, mitochondria (mito), and cytosolic (cytosol) fractions.

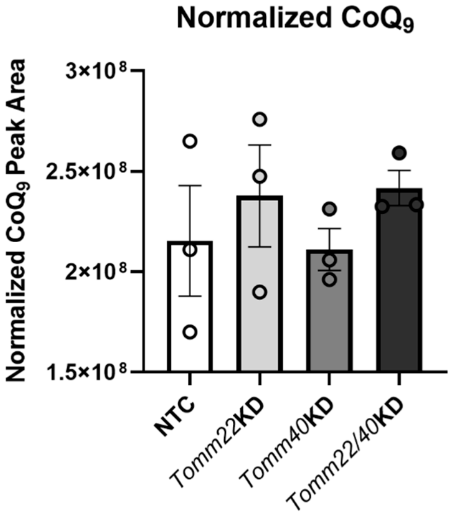

Figure S6. Effects of *Tomm40* and *Tomm22* knockdown on CoQ levels in C2C12 whole cell lysates. CoQ9 from whole cell lysates of NTC, *Tomm40, Tomm22*, and *Tomm22/40* KD C2C12 skeletal myotubes. All values were normalized to protein concentration. All numeric data represent mean ± SEM. Welch and Brown-Forsythe ANOVA, with Tukey's post-hoc test to identify differences between groups. (*n = 3* biological replicates)

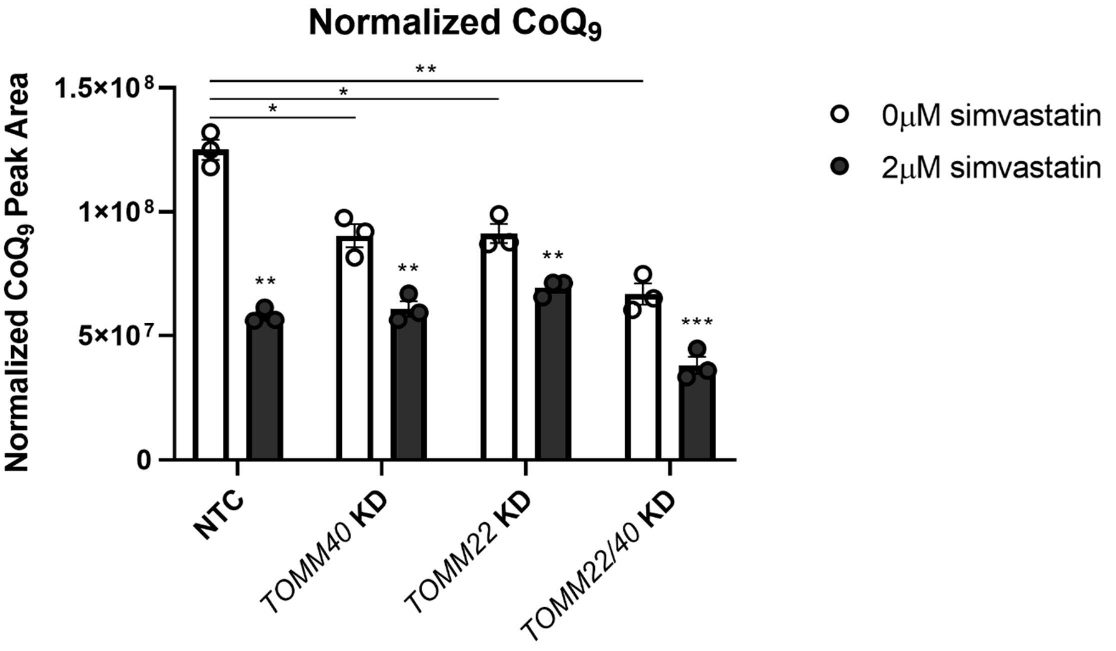

Figure S7. Simvastatin treatment further decreases CoQ levels in *Tomm40* and *Tomm22* KD C2C12 mitochondria. (A)Total CoQ_9 from isolated mitochondria of NTC, *Tomm40, Tomm22*, and *Tomm22/40* KD C2C12 skeletal myotubes treated with simvastatin (2 µM). All values were normalized to protein concentration. All numeric data represent mean ± SEM. **p<0.05*, ***p<0.01*, ****p<0.001*, *****p<0.0001* vs. 0µM simvastatin by Welch and Brown-Forsythe ANOVA, with Tukey's post-hoc test to identify differences between groups. (*n = 3* biological replicates)

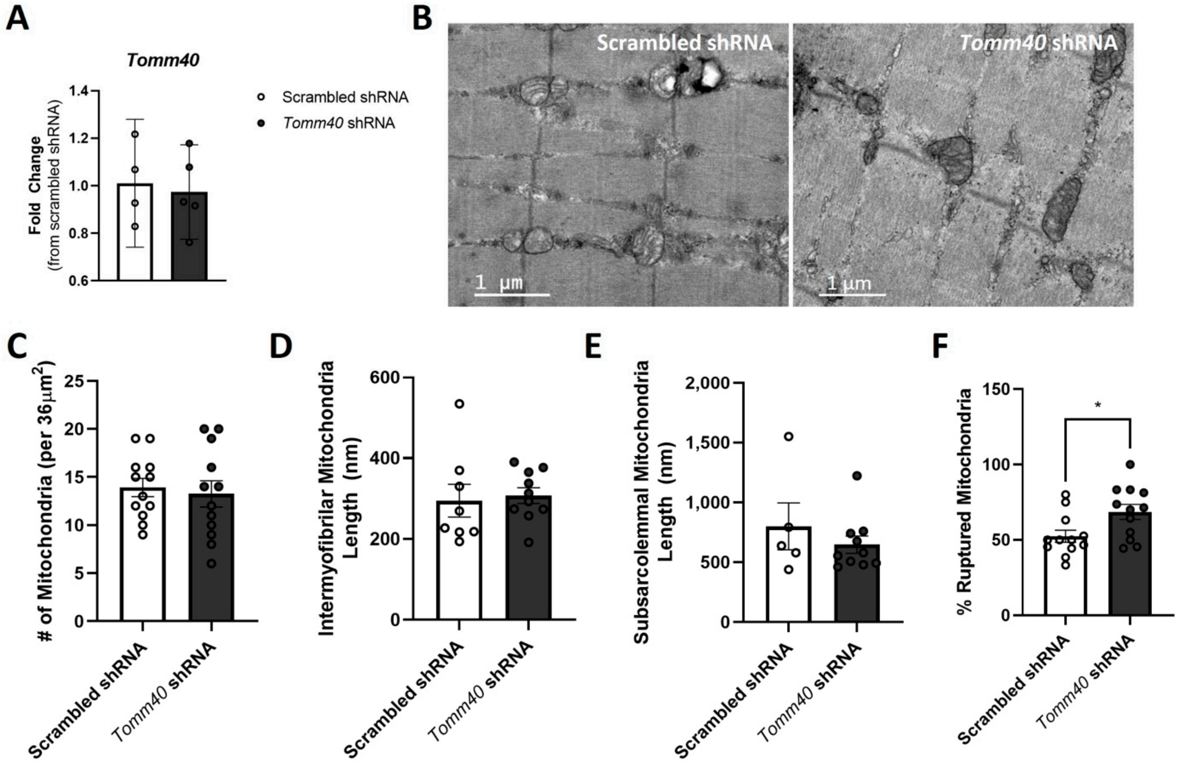

Figure S8. Female C57BL/6J mice injected with AAV8-*Tomm40* shRNA do not show knockdown of *Tomm40* or differences in mitochondrial number and morphology, compared to scrambled shRNA control. (A) Female mice IP injected with AAV8 scrambled vs. *Tomm40* shRNA do not show difference in *Tomm40* mRNA transcripts in gastrocnemius muscle tissues by qPCR ($n = 6$/group). (B) Representative TEM images of gastrocnemius muscle samples from scrambled vs. *Tomm40* shRNA ($n = 6$/group) female mice. (C) Bar graph represents average number of mitochondria within a surface area of 36 μm^2, (D) length of intermyofibrillar mitochondria (as seen in the TEM images), (E) length of sarcolemnal mitochondria (not shown), and (F) percent of ruptured mitochondria per 36 μm^2. All numeric data represent mean ± SEM. *$p<0.05$ vs. scrambled shRNA by Student's t-test.

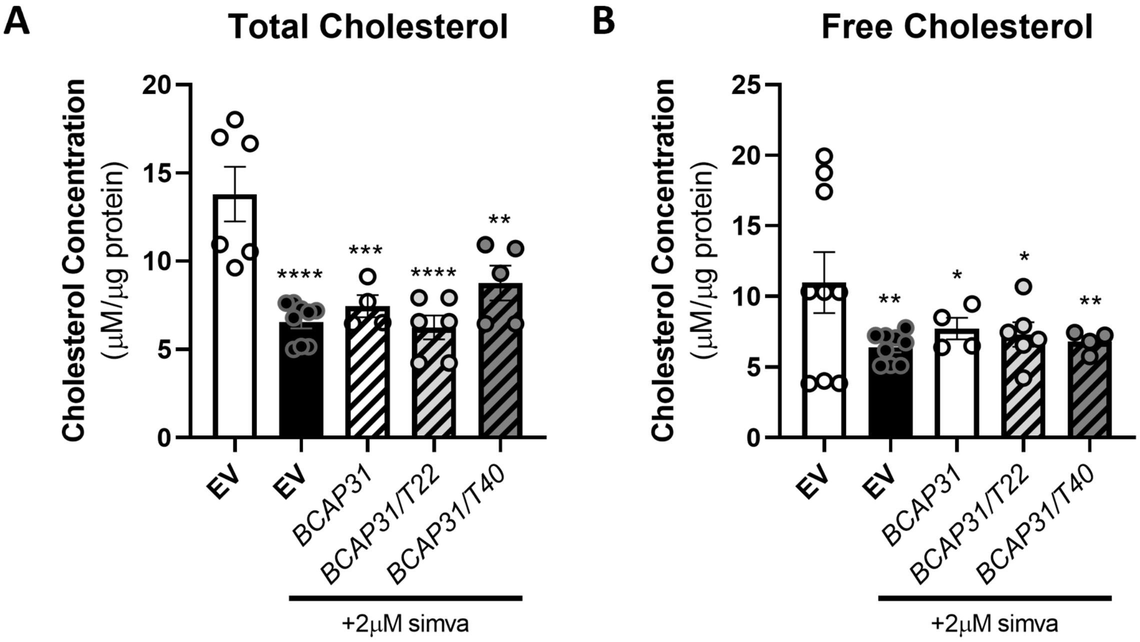

Figure S9. BCAP31 and TOMM40 or TOMM22 overexpression does not rescue intracellular cholesterol levels in simvastatin-treated hSkMC myotubes. (A) Total and (B) free cholesterol were quantified in mitochondria of C2C12 myotubes using Amplex Red Cholesterol Assay (*n = 6-12* biological replicates). All numeric data represent mean ± SEM. *$p<0.05$, **$p<0.01$, ***$p<0.001$, ****$p<0.0001$ vs. EV (without statin) by one-way ANOVA, with Tukey's post-hoc test to identify differences between groups.

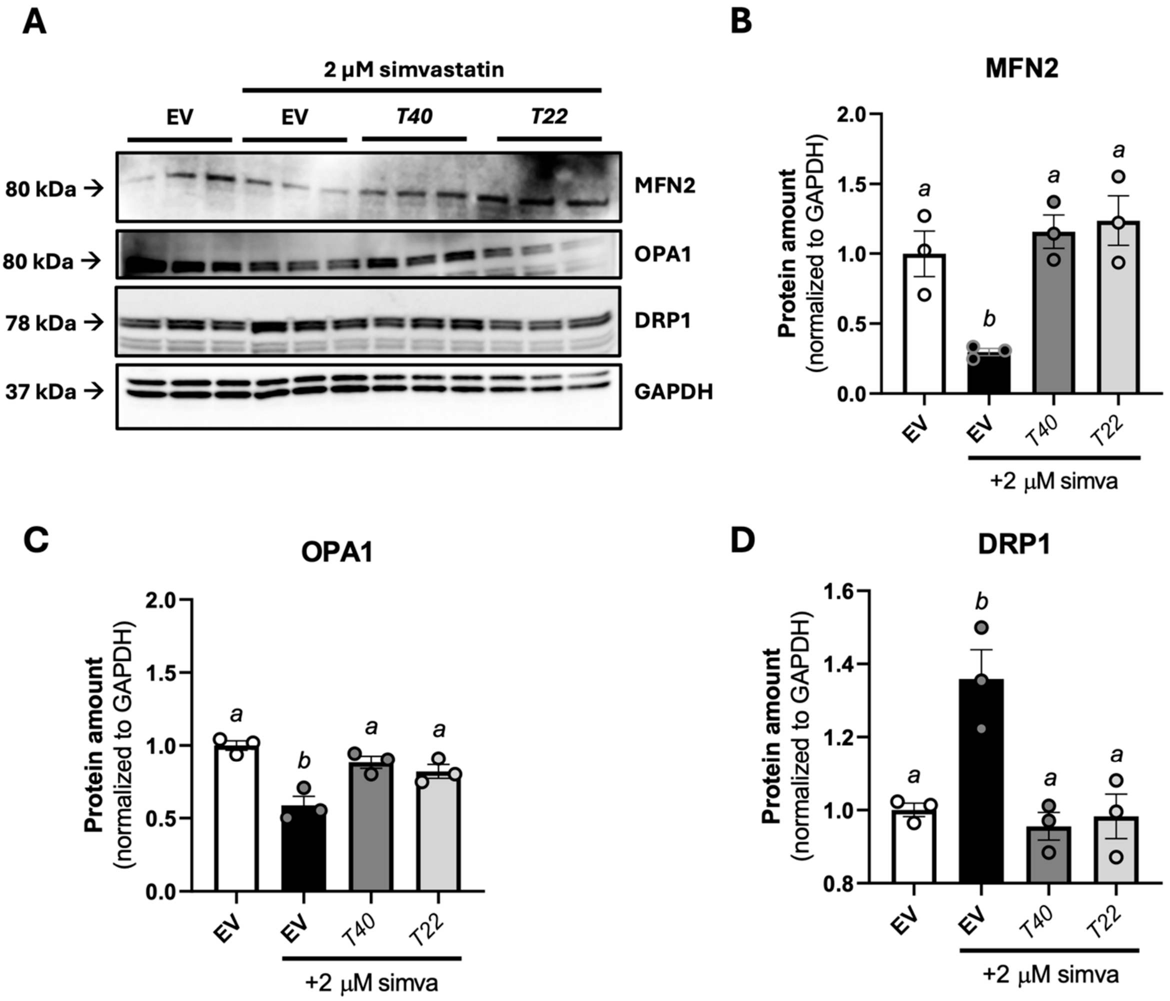

Figure S10. Overexpressing *TOMM40* or *TOMM22* rescues markers of mitochondrial dynamics in simvastatin-treated primary hSkMC skeletal myotubes. (A) Representative western blot and relative protein amount of (B) MFN2 and (C) OPA1 (fusion markers), and (D) DRP1 (fission marker) protein expression in EV or 2 μM simvastatin + EV, *T22, T40* primary hSkMC myotubes. All numeric data represent mean ± SEM. $p<0.05$ for *a* vs. *b* by two-way ANOVA, with Sidak's multiple comparisons test. ($n = 3$ biological replicates)

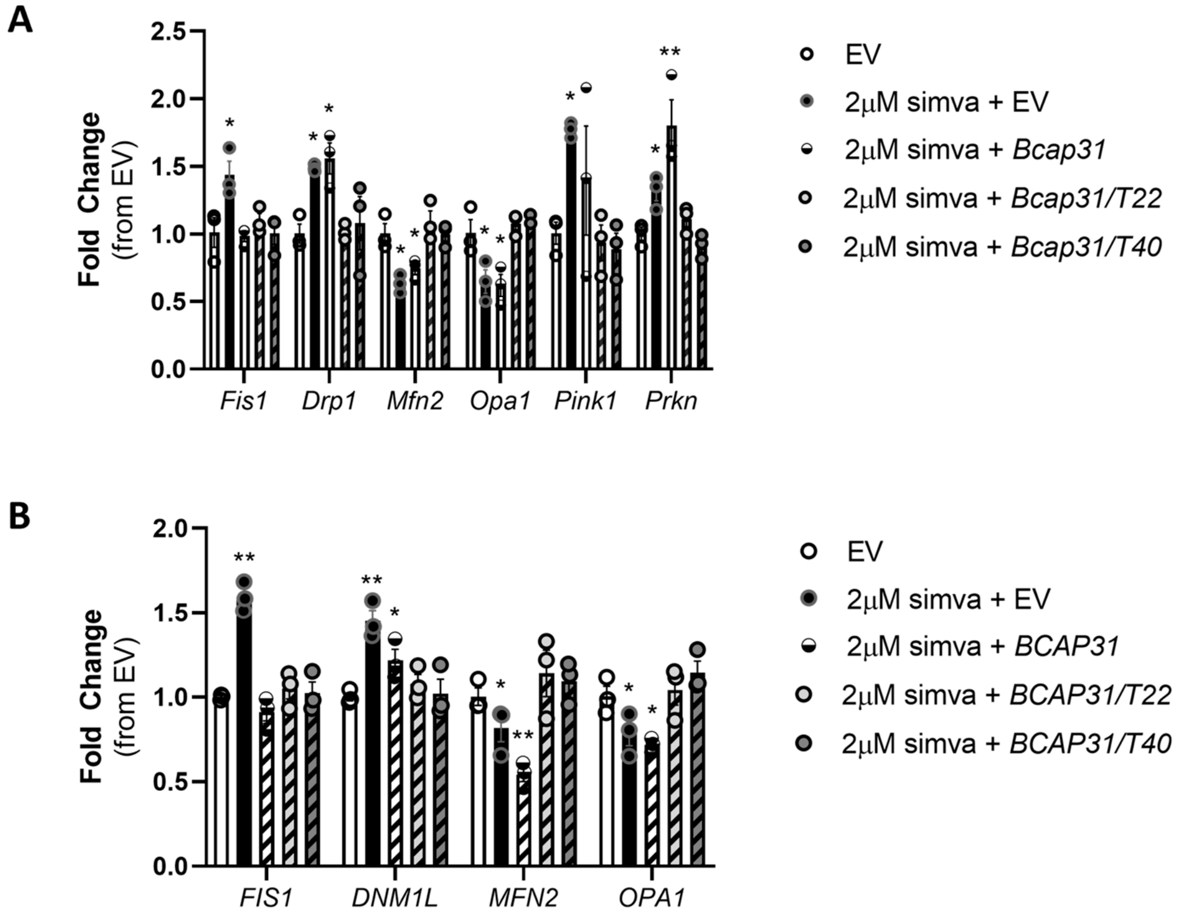

Figure S11. Overexpression of *BCAP31* recues *FIS1* gene expression in simvastatin-treated skeletal myotubes. (A) Mitochondrial fission (*Fis1/FIS1, Drp1/DNM1L*) and fusion (*Mfn2/MFN2, Opa1/OPA1*) markers were quantified by qPCR in EV and 2 μM simvastatin + EV, *Bcap31*, Bcap31/*T22,* and *Bcap31/T40* overexpressing C2C12 cells. (B) The same experiment was conducted as in (A) but with hSkMC cells. (*n=3* biological replicates). All graphical and numeric data represent mean ± SEM. **p<0.05*, ***p<0.01* vs. EV (without statin) by one-way ANOVA, with Tukey's post-hoc test to identify differences between groups.

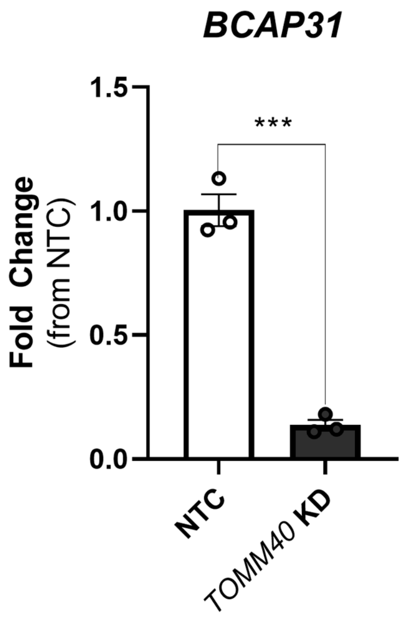

Figure S12. *TOMM40* knockdown downregulates *BCAP31* gene expression in hSkMC myotubes. mRNA transcripts show a reduction by ~85% by qPCR of *BCAP31* in differentiated hSkMC myotubes transfected with *TOMM40* siRNA for 48 hrs. ($n=3$ biological replicates) Graphical data represent mean ± SEM. ***$p<0.001$ vs. NTC by Student's t-test.
